# Host genotype and age shape the leaf and root microbiomes of a wild perennial plant

**DOI:** 10.1038/ncomms12151

**Published:** 2016-07-12

**Authors:** Maggie R. Wagner, Derek S Lundberg, Tijana G. del Rio, Susannah G. Tringe, Jeffery L. Dangl, Thomas Mitchell-Olds

**Affiliations:** 1 Program in Genetics and Genomics, Department of Biology, Duke University, Durham, North Carolina 27708, USA; 2 Department of Biology, Curriculum in Genetics and Molecular Biology, Carolina Center for Genome Sciences, University of North Carolina, Chapel Hill, North Carolina 27599, USA; 3 Department of Energy Joint Genome Institute, Walnut Creek, California 94598, USA; 4 Howard Hughes Medical Institute, Department of Microbiology and Immunology, University of North Carolina, Chapel Hill, North Carolina 27599, USA

## Abstract

Bacteria living on and in leaves and roots influence many aspects of plant health, so the extent of a plant's genetic control over its microbiota is of great interest to crop breeders and evolutionary biologists. Laboratory-based studies, because they poorly simulate true environmental heterogeneity, may misestimate or totally miss the influence of certain host genes on the microbiome. Here we report a large-scale field experiment to disentangle the effects of genotype, environment, age and year of harvest on bacterial communities associated with leaves and roots of *Boechera stricta* (Brassicaceae), a perennial wild mustard. Host genetic control of the microbiome is evident in leaves but not roots, and varies substantially among sites. Microbiome composition also shifts as plants age. Furthermore, a large proportion of leaf bacterial groups are shared with roots, suggesting inoculation from soil. Our results demonstrate how genotype-by-environment interactions contribute to the complexity of microbiome assembly in natural environments.

## Abstract (web summary)

Laboratory-based studies of the effect of plant genotype on plant microbiome composition often do not consider the influence of environmental heterogeneity. Here, Wagner *et al*. use a large-scale field experiment to assess the extent of host genetic and environmental factors on the microbiome of *Boechera stricta*.

## Graphical abstract

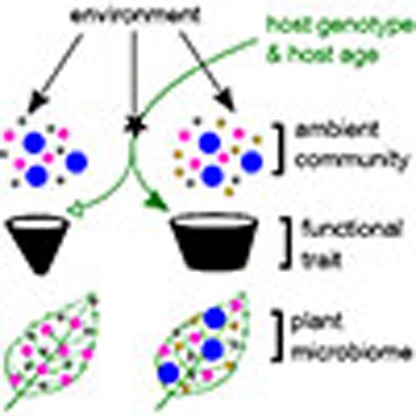

Bacteria can have wide-ranging effects on the success of their host plants, influencing plant processes such as disease resistance^1^,^2^,^3^, drought tolerance^4^, life cycle phenology^5^,^6^ and overall vigour^7^. The implications of plant–microbe interactions for agriculture^8^ and the ecology and evolution of wild plants^4^,^5^,^6^ have stimulated great interest in the factors that shape plant-associated microbiota. By treating the microbiome as a complex plant trait, we can apply quantitative genetic tools to disentangle multiple sources of variation in plant-associated bacterial communities^9^,^10^.

The extent of plant genetic control over bacterial communities is of interest to crop breeders and evolutionary biologists, because heritability of the microbiome determines whether it can evolve (as part of the host's extended phenotype) in response to selection on host plants^9^,^10^,^11^,^12^,^13^,^14^. Laboratory studies have confirmed that intraspecific genetic variation can alter endophyte communities of the root and rhizosphere (the thin region of soil permeated by root exudates)^15^,^16^,^17^,^18^ as well as leaf surfaces and interiors^19^,^20^.

Laboratory settings eliminate natural exogenous stimuli that drive plasticity of complex phenotypes, potentially including plant traits that underlie microbiome assembly. As a result, laboratory-based studies may overestimate the influence of certain plant genes on the microbiome composition in natural environments, while simultaneously failing to identify important loci with context-dependent functions^21^. Even in complex natural environments, the same problem arises when microbiomes are studied only within one field site or 1 year^20^. In agroecosystems and wild habitats, biotic and abiotic influences that could overwhelm, amplify, or mask the effects of host genes often vary between locations and years, resulting in context-dependent expression of genetic variation for functional traits that could shape the microbiome. For instance, quantitative trait locus mapping of field-grown maize exposed to low and high levels of ultraviolet-B radiation revealed multiple loci that controlled bacterial epiphyte diversity only under high ultraviolet-B, in addition to several loci with environment-independent (constitutive) effects^22^. Such targeted experiments are useful for studying the genes underlying microbiome responses to specific environmental stimuli. However, genetic variation for plastic responses to the suite of environmental factors that differentiate natural environments and agroecosystems should also be assessed^23^.

Another understudied potential source of microbiome variation is host age, which can affect expression of plant functional traits that influence the microbiome (for example, defensive chemistry^15^,^24^,^25^). Previous studies reported strong changes in phyllosphere (above ground) microbial communities over the course of a growing season^26^,^27^,^28^. For annuals, these patterns describe the microbiome throughout the life of the plant; but for perennials each growing season represents only a fraction of the plant's lifetime. Longer-term studies of trees have reported phyllosphere changes between years^27^,^29^, but could not separate effects of plant age from interannual variation. A more targeted experimental design is needed to learn how plant-associated microbial communities are maintained in aging perennial plants in the face of temporal variation.

We conducted a multi-year, large-scale field experiment using the short-lived perennial mustard *Boechera stricta*^30^ to disentangle the effects of genes, environment, and host age on plant-associated microbial communities. Because *B. stricta* is naturally inbreeding^31^, siblings produced by self-fertilization of wild accessions are nearly clonal, enabling replication of genotypes across multiple environments. The species has wide genetic diversity, and accessions from distinct populations are often highly divergent^31^. Many *B. stricta* populations and habitats are largely undisturbed throughout the species' range in western North America, allowing study of the plants in environments where they, and their associated microbes, have been evolving for thousands of years^30^.

We focus on the following questions: first, how do bacterial communities change over the lifetimes of perennial plants? Second, how much do plant genotype and interactions of plant genotype with the local environment contribute to microbiome variation? Third, do above- and below-ground organs show similar patterns of genetic control over their resident bacterial communities? Here we show that root microbiomes change as plants age from 2 to 4 years old; the host genotype effect on the phyllosphere microbiome is environment-dependent; and despite strong overlap in community membership, leaf and root microbiomes are shaped by different sources of variation.

## Results

### Leaves and roots harbour distinct microbiomes

**Figure 1:**
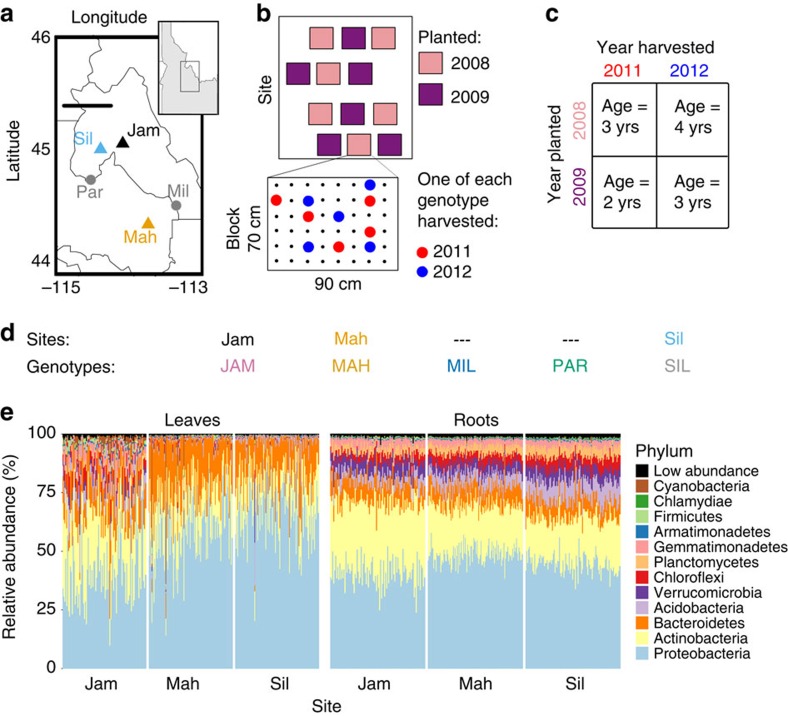
Summary of experimental design and analysis. (**a**) Map of the study region in central Idaho, USA (map data from the R package ‘maps'^70^). The five genotypes used in this experiment were collected from the five *B. stricta* populations shown. We collected seeds from 8–10 individual *B. stricta* plants from each population, for a total of 48 accessions or genetic ‘lines'. For our analyses these lines were grouped back into five ‘genotypes' corresponding to the populations from which their ancestors were collected. The populations marked with triangles correspond to the ‘sites' of the three common gardens where the experiment took place. Scale bar, 50 km. (**b**) Schematic representation of common garden layout. Each garden contained six replicated, randomized blocks per planting cohort (2008 and 2009). Each block contained one replicate of each ‘line', for a total of 8–10 replicates per ‘genotype'. In both 2011 and 2012, one individual of each genotype was haphazardly chosen for destructive sampling in each block. (**c**) A temporally staggered planting/harvesting design disentangled the effects of plant age and year of observation. (**d**) Abbreviations and colour codes are shown for the five genotypes and three sites featured in this study. (**e**) The relative abundances of major phyla are shown for each leaf or root sample.

We transplanted replicates of 48 *B. stricta* lineages (descended from accessions from five natural populations) into five common gardens in the native range in central Idaho, USA (Methods; Fig. 1a). Replicated, randomized experimental blocks within field sites (Fig. 1b) and independent lines within genotypes allowed us to assess multiple scales of environmental and genetic variation, respectively, using nested random effects in our statistical models. Identical cohorts were planted in 2008 and 2009; representative subsets of the surviving plants were destructively harvested in 2011 and 2012 for root and leaf samples from hosts aged 2, 3 or 4 years (Fig. 1c). This temporally staggered experimental design allowed us to test the effect of plant age while controlling for variation between years. We used several methods for statistical support: linear mixed models (LMMs) to predict α-diversity (within-sample diversity) and independent components of β-diversity (between-sample diversity) while accounting for our hierarchical experimental design; and negative binomial models (NBMs) to test for enrichment/depletion of individual bacterial taxa^32^,^33^. Our variance-partitioning model tests for effects of host age, host genotype and year on microbiome communities, while age-by-site, genotype-by-site and year-by-site interaction terms describe how the distinct bacterial communities at different common gardens respond differently to each of these factors.

Poor survival in two sites (‘Mil' and ‘Par', probably due to drought and erosion, respectively) unbalanced the experiment (Supplementary Table 1 and Supplementary Fig. 1); therefore, we only analysed the data from the remaining three sites (Fig. 1a). We quantified bacterial communities in 21 bulk soil, 306 whole-leaf and 310 whole-root samples of *B. stricta* by sequencing the V4 region of the 16S rRNA gene (Methods). Previous analyses showed that these bulk soil communities differed among field sites but not between years^6^. Each leaf sample comprised both epiphyte and endophyte DNA, and each water-washed root sample comprised both rhizoplane (root surface) and endophyte DNA. These 616 samples represented 440 individual plants across three common gardens (sites), 36 experimental blocks (12 nested within each site), five genotypes (each encompassing 8–10 genetic lines, to represent additional variation within each genotype), 2 years of observation and three age groups (Fig. 1). The final data set included 27,763±15,790 observations per sample (mean±1 s.d.), comprising 3,718 operational taxonomic units (OTUs) after we removed contaminants and non-reproducible OTUs (see Methods)^16^ and corrected counts for 16S rRNA gene copy number variation^34^. Distributions of OTU counts were highly positive skewed, especially in leaves (Supplementary Fig. 2), where one OTU (*Sphingomonas sp.*) accounted for 36% of all observations. Both leaf and root communities consisted largely of Proteobacteria, Actinobacteria and Bacteroidetes (Fig. 1e), but were distinguished from each other and from bulk soil by their most common families (Supplementary Tables 2–4 and Supplementary Fig. 1).

### Habitat and year strongly influenced microbiomes

**Figure 2:**
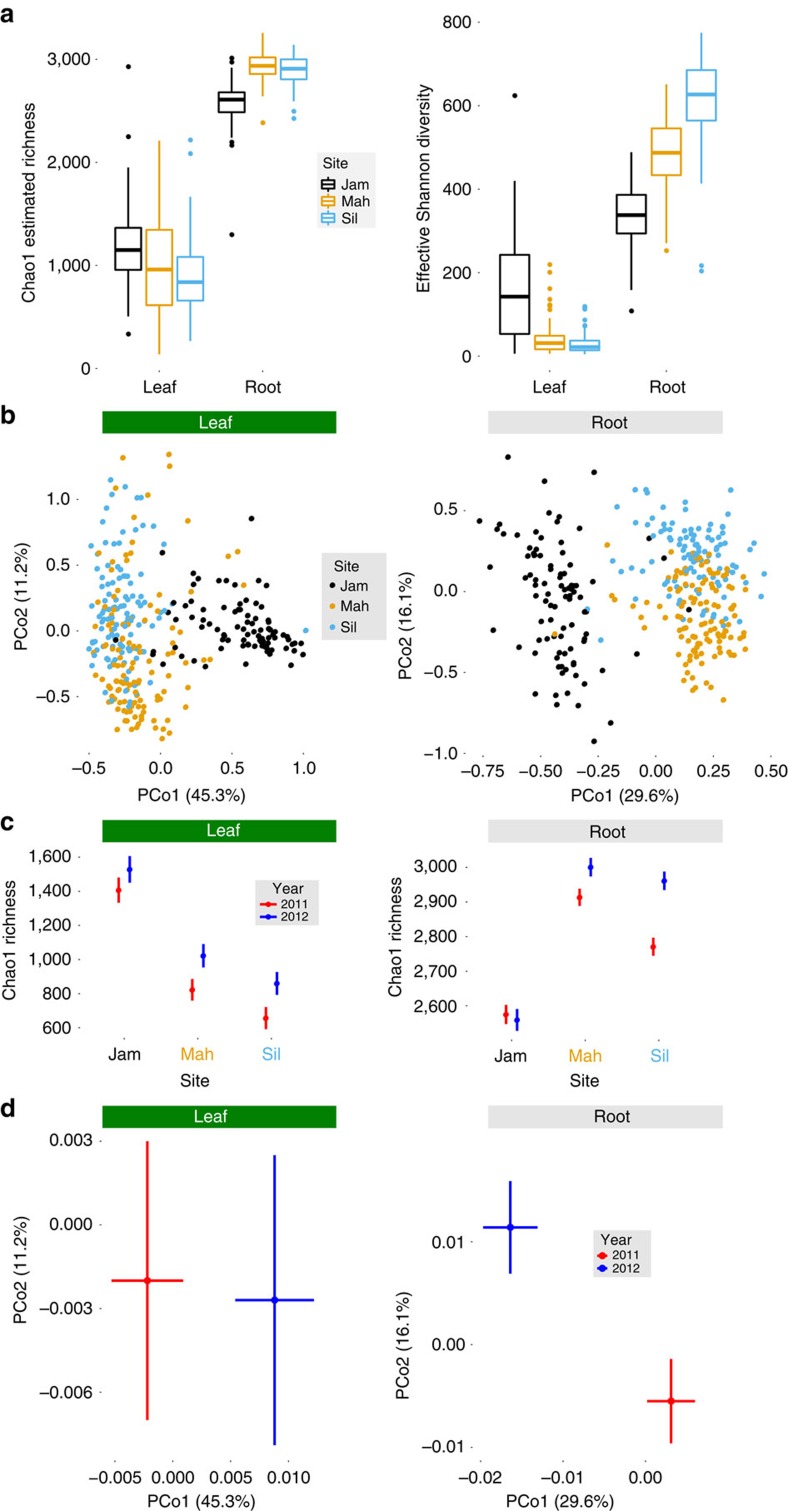
Habitats differ strongly in richness and composition of leaf- and root-associated bacterial communities. Sample sizes are *N*=306 for leaves and *N*=310 for roots. (**a**) Mean Chao1 richness and effective Shannon diversity (*e*^Shannon entropy^) differed among field sites for both roots and leaves (ANOVA, *P*<1.5e−11; detailed statistics are found in Table 1). The bottom and top edges of the boxes mark the 25th and 75th percentiles (that is, first and third quartiles). The horizontal line within the box denotes the median. Whiskers mark the range of the data excluding outliers that fell more than 1.5 times the interquartile range below the first quartile or above the third quartile (dots). (**b**) PCoA of weighted UniFrac distances reveals that field site is a major source of bacterial community variation in both leaves and roots (ANOVA, *P*<9e−7; detailed statistics in Table 2). Similar patterns result from PCoA of unweighted UniFrac distances, which only considers presence/absence of OTUs (Supplementary Fig. 4). (**c**) α-Diversity increased between 2011 and 2012 at most sites (ANOVA, *P*<0.05; detailed statistics in Table 1). Least-squares mean Chao1 richness is plotted for each year and each site; error bars represent 1 s.e.m. (**d**) Microbiome composition changed moderately between 2011 and 2012 (ANOVA, *P*=0.055 in leaves, *P*<0.01 in roots; detailed statistics in Table 2). Least-squares mean PCo1 and PCo2 are plotted with s.e.m. to show effects of year while controlling for other sources of variation using LMMs.

The three undisturbed *B. stricta* habitats that we used as field sites (Jackass Meadow, abbreviated ‘Jam' in the figures; Mahogany Valley [Mah]; and Silver Creek [Sil]) were separated by 26–92 km, ranged in elevation from 1,812 m (Silver Creek) to 2,676 m (Jackass Meadow), and varied in moisture, temperature, plant diversity and soil nutrition (Fig. 1a and Supplementary Fig. 3). Previous work showed that soils from these sites harbour distinct bacterial communities^6^; our experiment confirmed that bacterial communities inhabiting *B. stricta* leaves and roots also distinguish these habitats (Fig. 2 and Supplementary Fig. 1).

We found that α-diversity differed among sites, and principal coordinates analysis (PCoA) of weighted UniFrac distances^35^ demonstrated that field site also was the primary source of β-diversity (Fig. 2a,b and Tables 1 and 2). In both organs, the primary axis of variation distinguished the Jackass Meadow microbiota from the other sites, while the secondary axis of variation separated Mahogany Valley from Silver Creek, particularly in roots (Fig. 2b). This pattern was clear in the ordination of unweighted UniFrac distances as well, suggesting that the large number of rare OTUs also contributed to differences among habitats^35^ (Supplementary Fig. 4).

We also modelled the three major PCoA axes separately using analysis of variance (ANOVA) of LMMs, because PCoA axes represent components of variation that vary independently of each other and may be shaped by different sets of explanatory factors. The top three axes of the weighted UniFrac PCoA represented 56% of all variation in roots, and 63% in leaves (Supplementary Fig. 5). Field site predicted all major axes of variation in both leaves and roots (Table 2). Bacterial communities were similar at the metre scale (among blocks within sites; Tables 1 and 2), except perhaps in the presence of rare root OTUs, as suggested by unweighted UniFrac analysis (Supplementary Table 5).

**Figure 3:**
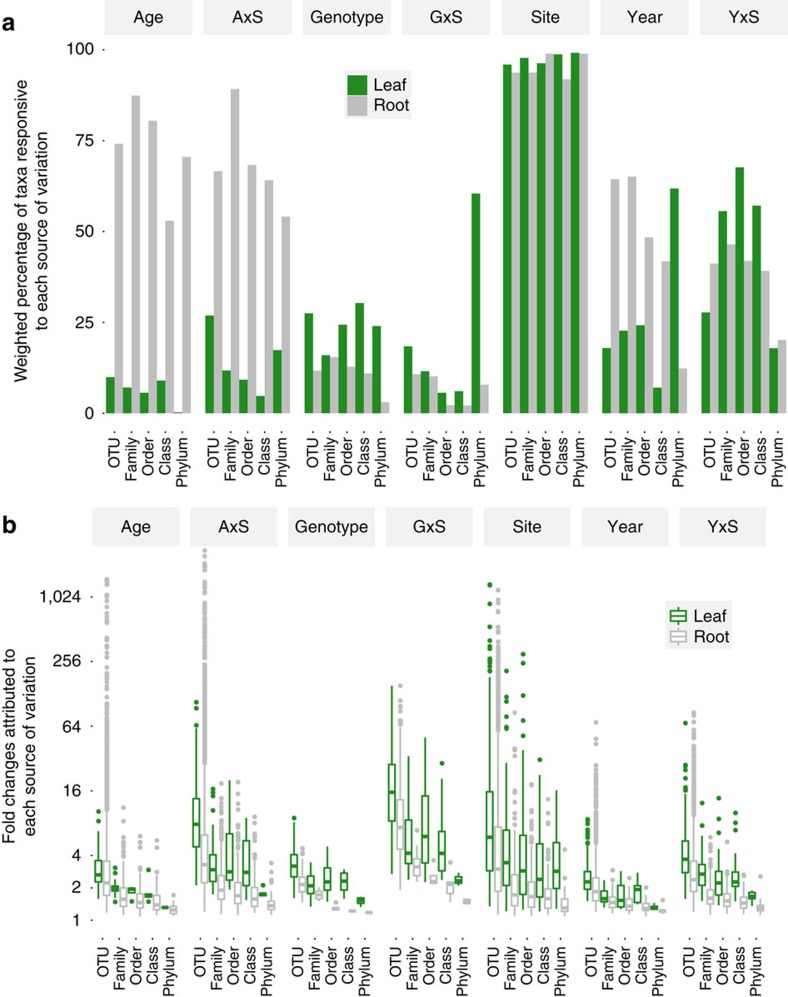
Individual bacterial taxa at multiple taxonomic levels are sensitive to several interacting factors. Abundances of bacterial OTUs, families, orders, classes and phyla were individually modelled using NBMs. Sample sizes are *N*=306 for leaves and *N*=310 for roots. Significance was assessed using a Likelihood ratio test at *P*<0.05 after correction for multiple comparisons using Benjamini–Hochberg false discovery rate. ‘Geno'=genotype; ‘GxS'=genotype-by-site interactions; ‘AxS'=age-by-site interactions; ‘YxS'=year-by-site interactions. (**a**) Bar plots show the total relative abundance of bacterial taxa predicted by each source of variation (top horizontal axis) at multiple taxonomic levels (bottom horizontal axis), in variance-stabilized NBMs. (**b**) Effect sizes (fold changes between experimental groups for each term on the top horizontal axis) are plotted for all statistically significant pairwise contrasts predicted by variance-stabilized NBMs (Wald test, *P*<0.05). The bottom and top edges of the boxes mark the 25th and 75th percentiles (that is, first and third quartiles). The horizontal line within the box denotes the median. Whiskers mark the range of the data excluding outliers (green or grey dots) that fell more than 1.5 times the interquartile range below the first quartile or above the third quartile. To improve readability of the plot, the vertical axis was truncated at 2^11^, concealing seven extreme outliers: all were root-associated OTUs with extreme changes in abundance due to age-by-site interactions.

We next investigated abundance patterns of individual OTUs, families, orders, classes and phyla. Unlike α-diversity and β-diversity metrics, which are continuous and approximately normal variables that can be modelled using LMMs, individual taxon abundances are discrete count data resembling a Poisson or negative binomial distribution. Therefore, we assessed taxon enrichment/depletion using NBMs of untransformed counts^32^,^33^. For these analyses, we considered only OTUs whose total abundances were at least 10% of the mean OTU abundance, which accounted for >98% of all observations. Although most OTUs were present at all sites (Supplementary Fig. 6), they were not equally abundant at all sites (Fig. 3a and Supplementary Data 1). Enrichments and depletions of taxa between pairs of sites were very common and relatively strong: the median statistically significant fold change for an OTU between two sites (averaged across all genotypes, age groups and years) was 5.9 in leaves and 3.0 in roots (Fig. 3b).

Bacterial microbiomes also varied between years. These temporal changes are not confounded with interannual differences in greenhouse conditions while seedlings were maturing, because individuals from both planting years were represented in each year of harvest (Fig. 1c). α-Diversity increased between 2011 and 2012 in both organs (Table 1 and Supplementary Fig. 7), although in roots this pattern was significantly diminished at one site (Fig. 2c). Year of harvest also contributed to PCoA separation of both leaf and root communities, indicating temporal changes in relative abundance of OTUs (Fig. 2d and Table 2); however, this effect was minor compared with the differences between sites. Many taxa shifted in abundance between years in both organs (Fig. 3a). Although fairly common, these temporal changes were weaker than changes attributable to other sources of variation (Fig. 3b). Bacterial communities of bulk soil samples did not differ significantly between years^6^, suggesting that the observed change in root communities may be driven by plant responses to other temporally varying environmental factors such as precipitation or temperature.

Together, these results demonstrate that in nature, both leaf and root microbiomes vary profoundly among sites and between years. This pattern was consistent from individual OTUs (Fig. 3a) to community-level measures of α and β diversities (Fig. 2 and Tables 1 and 2). Dissimilarity of plant microbiomes among habitats is a very common observation^23^,^36^,^37^,^38^,^39^, although not universal^40^. Such strong site effects likely reflect the influence of distinct soil pH and nutrient profiles on local bacterial communities (Supplementary Fig. 3), or other environmental factors such as water availability, temperature and ultraviolet radiation^41^,^42^. In addition to these likely biogeographic explanations, environmental heterogeneity could have affected leaf and root communities indirectly by causing plasticity of plant functional traits that underlie the microbiome. For instance, drought stress can alter cuticle thickness^43^, which in turn may affect microbial colonization^1^,^19^,^20^,^41^. Similarly, in our experiment, environmental differences among sites altered root glucosinolate defensive chemistry, which may affect root-associated bacteria (although bacteria themselves are part of a plant's environment and may have contributed to this phenotypic plasticity; Supplementary Note 1, Supplementary Table 6 and Supplementary Fig. 8)^6^,^15^. Therefore, variation in microbiomes of plants growing in multiple wild soils brought into the laboratory may not fully represent the variation that would be observed among the same plants growing in those soils in their natural settings.

### Bacterial communities changed as host plants aged

Our temporally staggered planting and harvesting design allowed us to test for effects of plant age after controlling for year-to-year changes (Fig. 1c). Because we germinated these plants in the greenhouse and then transferred them into common gardens as juveniles, plant age was confounded with microbial succession after a major habitat shift. This mostly affected roots, which received an initial microbial inoculum from the potting soil before exposure to natural soil communities. In contrast, the juvenile leaves that formed in the greenhouse fell off during the winter immediately following the autumn transplant, and were replaced by new spring growth. Furthermore, over the lifetime of the plant, these rosette leaves either senesced during the summer (to be regrown from the live root the following spring) or persisted between years. Therefore, our age measurement represents the plant as a whole, but not necessarily the sampled leaves themselves. In contrast, root samples generally comprised whole perennial root systems, with fractions of tissue ranging in age from new growth, to the same age as the plant, or even dead. Thus, systematic changes in the age distribution of root sub-sections are one possible mechanism for an effect of plant age on the root system as a whole.

To help distinguish true effects of host age from signals of post-transplant succession, we also sampled bulk soils and endogenous *B. stricta* plants (of unknown age) from natural populations in the vicinity of all five sites, resulting in 30 soil, 30 leaf and 23 root samples that had never been exposed to the greenhouse. Only 29 OTUs (of 3,718 total), with a combined relative abundance of 0.11% in all root samples, were present in our experimental plants but absent from these endogenous plants and wild soils (Supplementary Data 2 and Supplementary Fig. 9a). We removed these 29 ‘unnatural' OTUs (which were otherwise unremarkable; Supplementary Fig. 9b) from the data set before analysis. However, because several distinct bacterial strains may be lumped into a single OTU^44^, organisms persisting from the greenhouse might still be counted as ‘natural' OTUs that also were observed in the endogenous plants. Indeed, 84.5% of OTUs from endogenous plants and wild soils were also found in samples from the greenhouse (Supplementary Note 2). Limited resolution of intra-OTU variation is an inherent drawback of 16S rDNA profiling. Therefore, the transplants' similarity to endogenous plants, their dissimilarity to greenhouse controls and their strong microbiome divergence between field sites despite their origin in a common potting soil—not the presence/absence of particular OTUs—provide the best evidence that wild bacteria had largely replaced the initial greenhouse inoculum (Fig. 2b, Supplementary Fig. 10 and Supplementary Table 7). The bulk of the evidence suggests that microbiomes of our experimental plants were much more wild than not, and that host age effects can be partially, but not entirely, explained by the attrition of potting-soil bacteria (Supplementary Figs 10–11 and Supplementary Note 2). Thus, we infer that the initial potting-soil inoculum reached equilibrium with surrounding soil within 2 years of transplant, and the plants continued to fine-tune their root microbiota for another 1–2 years.

**Figure 4:**
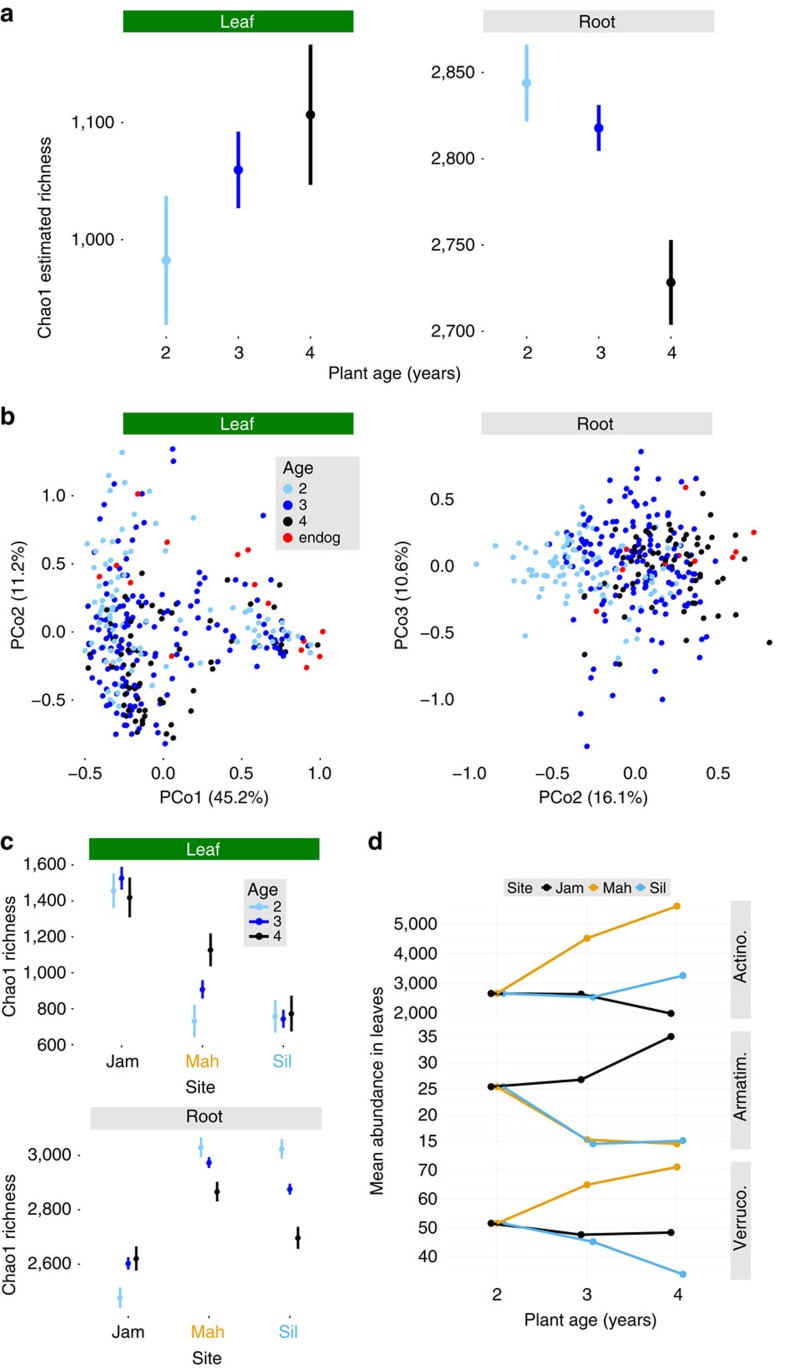
Leaf- and root- associated bacterial communities change as plants age. Detailed statistics for all tests are found in Tables 1 and 2. Sample sizes are *N*=306 for leaves and *N*=310 for roots. (**a**) Within-sample diversity declines with age in roots (ANOVA, F_2,57_=6.08; *P*=0.0081) but not in leaves (F_2,64_=1.11; *P*=0.67). Least-squares mean diversity estimates are plotted to show the effect of age after controlling for other sources of variation. Bars depict 1 s.e.m. (**b**) PCoA of weighted UniFrac distances between samples reveals that root bacterial community composition shifts over the lifetime of the plant. In roots, communities of experimental plants become more similar to those of endogenous plants, suggesting a role of succession after transplant (see Supplementary Note 2 for a detailed treatment of this hypothesis). Detailed statistics for the top three PCoA axes are found in Table 2. (**c**) Least-squares mean estimates of Chao1 richness are plotted for each age group in each site, illustrating how the distinct plant-associated bacterial communities in these habitats respond differently to host age. Leaves: F_4,63_=1.48; *P*=0.36. Roots: F_4,56_=7.85; *P*=8.6e−5. Error bars depict 1 s.e.m. (**d**) In leaves, the effect of plant age on the abundance of several phyla differs among sites (Likelihood ratio test, *P*<0.05 after Benjamini–Hochberg correction for multiple comparisons). Estimated mean abundances from NBMs are plotted for each age group at each site. ‘Actino.'=Actinobacteria; ‘Armatim.'=Armatimonadetes; ‘Verruco.'=Verrucomicrobia.

Leaf communities remained largely stable as host plants aged (Fig. 4a–c and Tables 1 and 2), although age-by-site interactions affected abundance of multiple taxa (Fig. 3). For instance, plant aging affected abundance of leaf-associated Actinobacteria, Armatimonadetes and Verrucomicrobia differently at various sites (Fig. 4d). Root α-diversity declined monotonically with plant age (Fig. 4a and Table 1). Age also shaped root β-diversity: root communities of older plants were more similar to those of endogenous plants, probably representing the replacement of potting-soil microbes by natural ones (Fig. 4b). This replacement process, along with true effects of plant age, impacted most bacterial taxa—including the phyla Acidobacteria, Armatimonadetes, Bacteroidetes, Chloroflexi, Firmicutes, Gemmatimonadetes, Planctomycetes and Proteobacteria (Fig. 3a, Supplementary Data 1 and Supplementary Note 2).

Thus, the microbiome of this perennial plant changed in both richness and composition over the host's lifetime. Although all plants were exposed to a common soil community as seedlings, within 2 years they had largely replaced those bacteria with the local endogenous communities of their respective field sites (Fig. 2). Root-associated communities continued to develop for years following this initial differentiation, and abundance of many leaf-associated taxa changed with host age as well, indicating that microbiome changes accompany the aging process even in perennials that are neither juvenile nor senescent.

### Host genotype shaped leaf but not root microbiomes

The five *B. stricta* genotypes used in this experiment originated from populations endemic to the three field sites analysed in this study, plus two other populations in the region corresponding to sites where high mortality prevented analysis of experimental transplants (Fig. 1a and Supplementary Table 1). Each genotype comprised 8–10 independent ‘lines' that were descended from separate accessions from the population of origin. Therefore, each line is more isogenic than its overarching genotype (in *B. stricta*, genetic variation is high between populations but low within populations^31^). In the LMMs, we treated the lines as random effects nested within genotypes.

**Figure 5:**
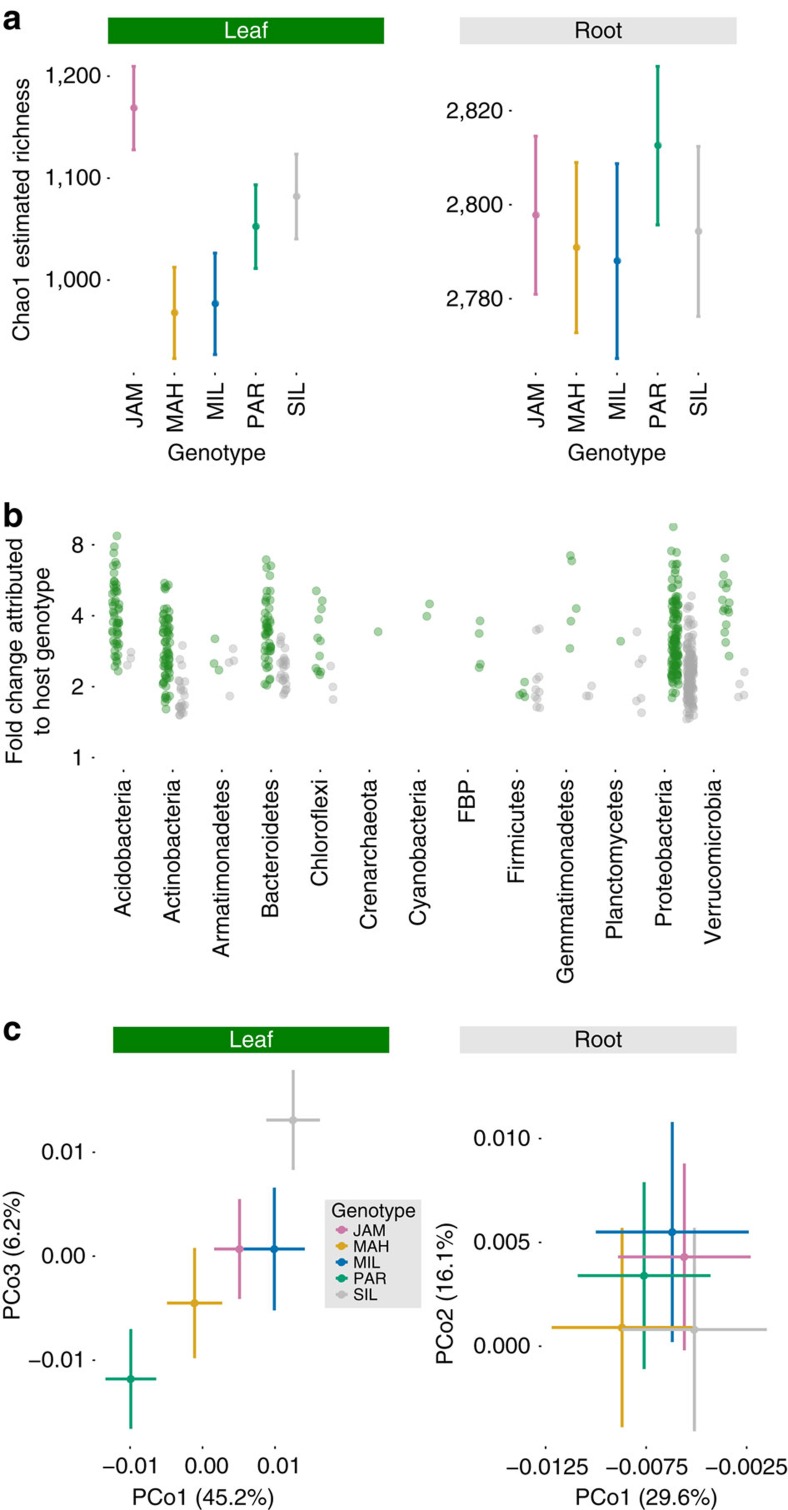
Host genotype shapes α and β diversities of the leaf microbiome but has weaker effects on root communities. The results shown here represent only the constitutive or environment-independent component of genetic variation, that is, genotype differences averaged across all sites. Sample sizes are *N*=306 for leaves and *N*=310 for roots. (**a**) Least-squares mean estimates of within-sample diversity are plotted for each plant genotype, after controlling for other sources of variation using linear mixed effects models. Leaves: F_4,265_=4.68, *P*=0.0023; Roots: F_4,261_=0.344, *P*=1. Bars depict 1 s.e.m. (**b**) Each point is an estimated differential abundance of one OTU between a pair of plant genotypes, plotted as a fold change (note log scale on vertical axis). Leaf OTUs are shown in green, root OTUs in grey. Differential abundance estimates and statistical significance were both generated using variance-stabilized NBMs that also controlled for site, year of observation, plant age, genotype-by-site interaction, age-by-site interaction and year-by-site interaction. Only statistically significant contrasts are shown (Wald test, *P*<0.05 after Benjamini–Hochberg correction for multiple comparisons). (**c**) Bacterial community composition separates by host plant genotype in weighted UniFrac ordination for leaves, but not roots. Least-squares mean PCoA coordinates are plotted to highlight the influence of host genotype after controlling for other sources of variation using LMMs. Detailed statistics for each PCoA axis are found in Table 2. Bars depict one standard error of the mean.

The main plant genotype effect, which describes the constitutive (that is, non-responsive to site) influence of host genetic variation averaged across all sites, years and ages, consistently influenced leaf-associated microbiota. Controlling for all other factors, species richness was 20% higher in leaves of plants with the *Jackass Meadow* (*JAM*) genotype, compared with the *Mill Creek* genotype (*MIL*; Fig. 5a). In contrast, plant genotype did not predict Shannon diversity, suggesting that rarer taxa (which are downweighted in Shannon diversity scores) may be more sensitive to host genotype than are common taxa (Supplementary Fig. 12 and Table 1). These rarer leaf taxa included Acidobacteria, Armatimonadetes, Verrucomicrobia and Gemmatimonadetes, plus several classes, orders, families and OTUs within thirteen diverse phyla that were differentially abundant among plant genotypes (Figs 5b and 3a, and Supplementary Data 1). These changes in leaf OTU abundance caused by host genotype were not as strong as those caused by site: the median size of a statistically significant genotype effect was a 3.2-fold change, compared with 5.9-fold change between sites (Fig. 3b).

Although the leaf taxa identified by NBMs as differentially abundant between plant genotypes represented only a modest proportion of the community (Fig. 3a), host genotype also affected the weighted UniFrac metric of β-diversity, which de-emphasizes rare taxa^35^ (Fig. 5c and Table 2). Even if enrichment of a particular common taxon is too subtle to be detected by individual regression, coordinated responses of multiple common taxa in response to the same source of variation may affect weighted UniFrac distances between these samples. Thus, host genotype influenced both common and rare leaf taxa, based on the complementary approaches of weighted UniFrac (Fig. 5c and Table 2), NBMs of individual counts (Fig. 3), and unweighted UniFrac (Supplementary Fig. 13 and Supplementary Table 5). Nonetheless, the plant genotype effect averaged across sites was relatively weak. In addition, fine-scale plant genetic variation (lines within genotypes) did not predict any metric of α-diversity or β-diversity (Tables 1 and 2 and Supplementary Table 5), indicating that genetic variation for the leaf microbiome primarily exists between genotypes from different populations of *B. stricta*, rather than between lines within populations. This corresponds with population genetic analyses in this species, where most genetic variation is found between populations^31^.

In contrast, plant genotype had a much weaker influence on root-associated bacterial communities. Host genotype did not predict any summary metrics of root α-diversity or β-diversity (Fig. 5a,c and Tables 1 and 2). Plant genotype affected abundance of root-associated bacterial taxa including 240 OTUs representing 10 different phyla (Fig. 5b and Supplementary Data 1); however, these were rare taxa that together contributed only a small portion of the community (Fig. 3a). Host genotype effects were less common in roots than leaves, and generally weaker; the median statistically significant OTU differential abundance between host genotypes was a 2.1-fold change, compared with 3.2-fold in leaves (Figs 5b and 3b).

**Figure 6:**
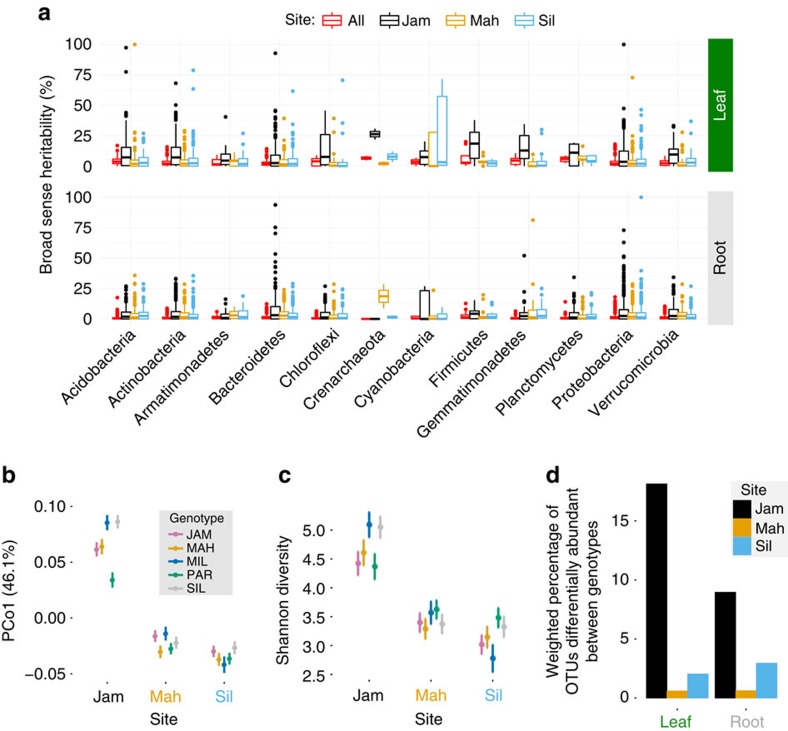
Host genetic control of the bacterial microbiome differs among habitats. Sample sizes are *N*=306 for leaves and *N*=310 for roots. (**a**) Estimates of broad-sense heritability (*H*^2^) of individual OTUs are plotted for leaves (top) and roots (bottom). The bottom and top edges of the boxes mark the 25th and 75th percentiles (that is, first and third quartiles). The horizontal line within the box denotes the median. Whiskers mark the range of the data excluding outliers that fell more than 1.5 times the interquartile range below the first quartile or above the third quartile (dots). (**b**) Between-sample diversity of the leaf microbiome is plotted as least-squares mean PCo1 of the weighted UniFrac distance for each plant genotype in each site, showing the genotype-by-site interaction after controlling for other sources of variation in a LMMs, including the constitutive effect of plant genotype and average site effects; F_8,257_=4.53, *P*=0.00011. Bars depict one standard error of the mean. (**c**) Least-squares mean leaf Shannon diversity is plotted for each genotype and each site, revealing site-dependent differences in the relative leaf community richness among host genotypes; F_8,261_=2.33, *P*=0.04. Bars show 1 s.e.m. (**d**) The total relative abundance of OTUs that were predicted by site-specific genotype effects in NBMs is shown for leaves and roots in each site (Wald test, *P*<0.05 after Benjamini–Hochberg correction for multiple comparisons).

In summary, averaged across all sites, plant genotypes differed in richness and composition of their leaf microbiota but genetic control of root communities was much weaker. This is consistent with observations that the *B. stricta* relative *Arabidopsis thaliana* has limited genetic influence over its root microbiome^16^,^17^. In both organs, host genetic control was weak compared to the variation contributed by differences among sites. Within-site heritability, however, was slightly higher than experiment-wide heritability (Fig. 6a), similar to the pattern seen in maize rhizosphere communities^23^. The genetic and phenotypic differences between these *B. stricta* genotypes are not yet well characterized; however, they at least vary in glucosinolate content (Supplementary Note 1), offering one plausible mechanism for the observed plant genetic variation in microbiome composition^15^. Further investigation of variation in host genotypes and phenotypes will be necessary to fully understand the functional basis of host microbiome control. Our results provide another^20^,^37^ example of intraspecific plant genetic variation for associated microbiota, although other studies have found little genotype effect^26^,^36^.

### The strongest genetic effects on microbes were site-specific

The genotype-by-site interaction term in our statistical models allowed us to describe how the genetic variation among our inbred plant genotypes mapped to the microbiome ‘phenotype' differently in each habitat^45^. These site-specific host genetic effects shaped the bacterial communities much more strongly than constitutive plant genetic variation averaged across all sites (Fig. 6). In leaves, host genotype-by-site interactions predicted the primary weighted UniFrac PCoA axis, indicating a strong, site-specific contribution of host genetic variation to β-diversity (Table 2 and Supplementary Table 5). Host genotype-by-site interactions predicted the abundance of Acidobacteria and Proteobacteria as well as multiple lower-level taxa including the Pseudomonadaceae (Fig. 3a and Supplementary Data 1). Although host genotype-by-site interactions predicted abundance of only a modest proportion of individual taxa, their effects were stronger than any other source of variation: the median change in leaf-associated OTU abundance due to a site-specific host genotype difference was 15.5-fold, compared with 3.2-fold for a host genotype effect averaged across sites, or the median 5.9-fold difference between pairs of sites (Fig. 3b). Furthermore, PCoA revealed that host genotypes separated more widely when interacting with site than when averaged across sites (compare PCo1 magnitude in Figs 6b and 5c). This strong host genotype-by-site interaction controlling weighted UniFrac distances between communities suggests that in addition to detectable effects on (mostly rare) individual taxa (Fig. 3a), site-specific host genetic variation also drives coordinated shifts in groups of common bacteria that together contribute to major principal coordinates of β-diversity.

In roots, host genotype-by-site interactions (like the main genotype effect) had little influence over community-level metrics of α-diversity or β-diversity (Fig. 3a, Tables 1 and 2 and Supplementary Table 5). A minority of root-associated taxa, including Burkholderiaceae, were affected by site-specific host genetic effects (Fig. 3a and Supplementary Data 1). As in leaves, these genotype-by-site interactions were also stronger than the site-independent genotype and site effects, with median changes in OTU abundance of 7.3-fold, 2.1-fold and 3.0-fold, respectively (Fig. 3b).

Several lines of evidence suggested that the influence of plant genetic variation on bacterial communities was amplified at Jackass Meadow compared with the other sites. LMMs of weighted UniFrac PCoA axes revealed that even after controlling for the site-independent plant genotype effect and other predictors, leaf communities showed elevated divergence among plant genotypes at Jackass Meadow (Fig. 6b). Furthermore, broad-sense heritability of individual OTUs across diverse bacterial phyla—in both leaves and roots—was greater at Jackass Meadow than at the other sites (Fig. 6a). Finally, in both organs, more OTUs were sensitive to host genotype at Jackass Meadow (Fig. 6d). This pattern could indicate that Jackass Meadow is a more homogeneous or less stressful environment than the other sites, thus decreasing the proportion of phenotypic variance attributable to environmental causes^46^. However, spatial variation in soil properties was not markedly less at Jackass Meadow than at the other sites (Supplementary Fig. 3; although note that because these data are from a single year they are not informative regarding temporal environmental stability, and furthermore many unmeasured environmental characters also distinguish these complex habitats). Alternatively, stronger influence of genetic variation might be expected if Jackass Meadow were ‘more' stressful than the other sites, because slightly deleterious genetic variation that is only expressed under unusual conditions may not have been previously available for purifying selection^47^,^48^. This explanation has somewhat better support, because environmental characteristics of Jackass Meadow diverge from the other sites along the major axis of variation (Supplementary Fig. 3). Furthermore, we observed rust infections of *B. stricta* exclusively at Jackass Meadow throughout the duration of this experiment (M.R.W. and T. M.-O., pers. obs.); genetic variation for fungal pathogen resistance could have pleiotropic effects on the bacterial microbiome^1^. Finally, polymorphism in genes that control trait plasticity in response to specific environmental stimuli can cause context-dependent patterns of genetic variation that are difficult to predict^49^,^50^. Despite the generally weak signal of host genotype on root communities in our study, the pattern of increased genetic effects at Jackass Meadow was salient in both roots and leaves (Fig. 6), raising the possibility that microbiomes in both organs may be influenced by host genetic variation with site-specific penetrance.

**Figure 7:**
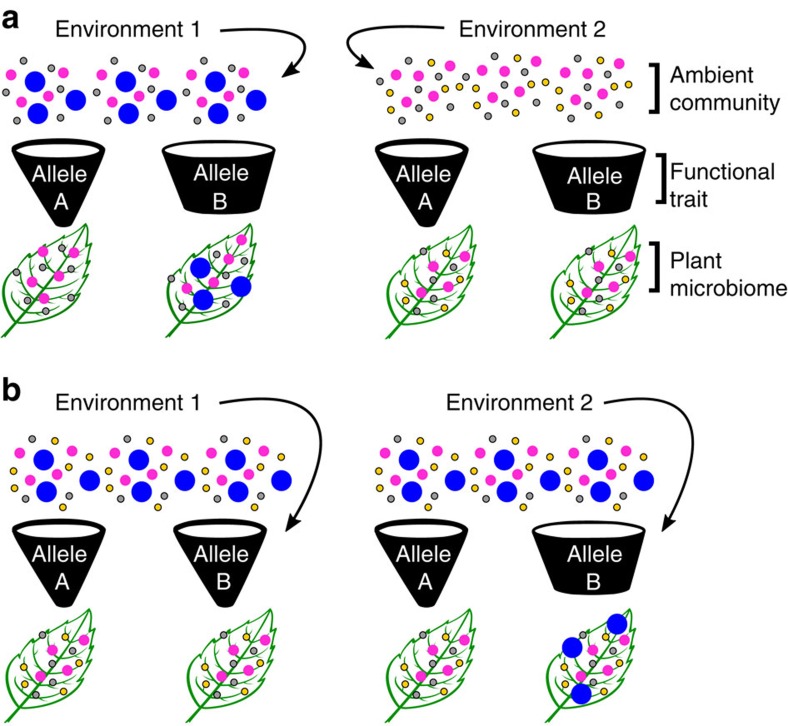
Multiple non-mutually exclusive hypothetical mechanisms could cause genotype–environment interactions for microbiome composition. Each panel shows the ambient microbial community for two environments (abstracted as coloured circles); the expressed phenotypes of two different host genotypes (abstracted as funnels that are labelled with the plant genotype); and the resulting microbiome of the genetically distinct plants in each environment. In **a** the genetic variant is expressed in both environments (that is, there is no phenotypic plasticity) but it affects only certain microbes, which are absent from Environment 2. As a result, genetic diversity for the trait is expressed in both environments, but genetic diversity for the microbiome is only observed in Environment 1. In **b**, environmental variation alters the phenotype expressed by plant genotype B, but not plant genotype A (that is, there is genetic variation for phenotypic plasticity, or a genotype–environment interaction for the functional trait). As a result, host genotype affects microbiome composition only in Environment 2, even though the ambient communities are identical in both environments.

Overall, our results show that environmental variation can alter the effect of plant genotype on the associated microbiota—host genetic variation underlying the microbiome is expressed differently in different sites. Host genotype-by-environment interactions have been shown to influence maize rhizosphere communities^23^; our study confirms that they also affect plant microbiomes in unmanaged natural habitats. One explanation is that host genetic variants could affect only certain groups of microbes, which may be present in differing quantities among the ambient communities in plant habitats (Fig. 7a). Another possible explanation is that plant genotypes differ in the extent of phenotypic plasticity^45^, resulting in site-specific patterns of host genetic variation in plant traits that in turn affect plant-associated microbial communities (Fig. 7b). To fully understand the genetic basis of plant control of the microbiome, it will be necessary to conduct genome-wide association studies and mutant experiments not only using diverse microbial inocula, but also under various realistic conditions such as drought stress and nutrient limitation. This is important for efforts to understand the evolution of plant–microbe interactions and to breed plants that form beneficial microbial associations^8^. The complication of genotype–environment interactions has long been recognized in both natural evolution and breeding programs for other agriculturally important traits^45^,^51^, and the microbiome appears to be no exception.

### Leaf and root microbiomes share many bacterial OTUs

**Figure 8:**
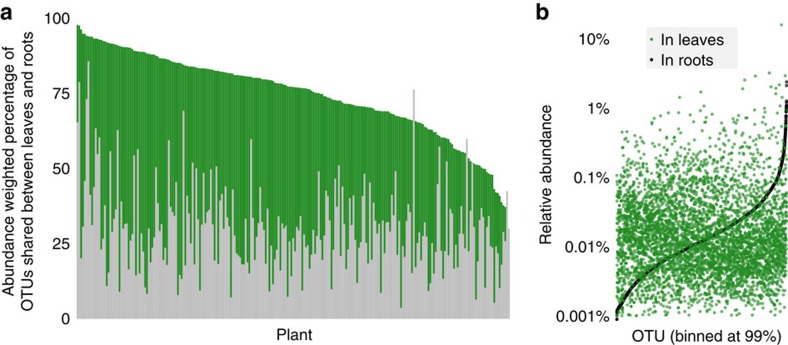
A large fraction of leaf OTUs were also observed within roots of the same plant. (**a**) Each bar shows the total fraction of leaf (green) and root (grey) OTUs (binned at 99% sequence similarity) belonging to the ‘shared set' of OTUs that were observed in both organs within one plant (*N*=237 plants). (**b**) Root OTUs from across the relative abundance spectrum contributed to the bacterial leaf microbiome. All root OTUs are plotted in increasing order of their relative abundance in roots (black dots). For each OTU that was also observed in leaves, its relative abundance in the leaf microbiome is plotted above or below it (green dots).

After removing extremely rare OTUs from the data set (see Methods), only seven OTUs were exclusively observed in leaves, and only 73 OTUs were exclusively observed in roots. These ‘organ-specific' OTUs were rarer than average, representing only 0.0006% and 0.3% of the leaf and root communities, respectively (Supplementary Fig. 14). To more stringently assess the degree of overlap between aboveground and belowground communities, we focused on the set of plants for which we had data from matched leaves and roots (*N*=237 plants) and reanalysed the raw reads to form OTUs based on a 99% sequence similarity threshold. On average, 74% (±14% s.d.) of all leaf-inhabiting OTUs (weighted by relative abundance) were also present in the root of the same plant (Fig. 8a). Furthermore, 93% of the OTUs that were present in both organs were also present in bulk soil—likely an underestimate due to our small number of bulk soil samples (*N*=30). This suggests that many *B. stricta* leaf epiphytes and endophytes may be derived from the soil. This sharing of OTUs between leaves and soil/roots may be a feature of low-growing plants (ours were usually 1–5 cm tall) which may be exposed to soil regularly through wind, rain splashes and crawling insects. This explanation is consistent with substantial inferred aboveground-belowground microbial overlap in grapes and *Arabidopsis*^52^,^53^, and minimal overlap reported for tropical trees^54^. Leaves at Jackass Meadow harboured a higher proportion of phyla that were much more common in root samples (Fig. 1d)—this may have been caused by drier, dustier, windier conditions at that high-elevation site resulting in soil deposition on leaves.

The set of shared OTUs represented a much smaller proportion of the more diverse root community. On average, 30.4% (±14% s.d.) of root OTUs were also observed in leaves of the same plant (Fig. 8a). Although 50.3% of shared OTUs were rarer in leaves than roots on average, many persisted aboveground at high or medium relative abundance, suggesting that bacterial taxa from across the relative abundance spectrum in roots are also capable of colonizing leaves and flourishing in the phyllosphere (Fig. 8b). Shared OTU relative abundances in the two organs were positively, but weakly, correlated (analysis of covariance, *P*<3e−16, *R*^2^<0.004; Fig. 8b). Together, these data suggest that leaves and roots harbour different but overlapping subsets of bacterial taxa, but relative abundances in the root are not good predictors of relative abundances of those shared taxa in the leaf. This is consistent with recent reports that bacteria isolated from roots of *A. thaliana* can colonize leaves (and vice versa), although strains are generally better adapted to one organ or the other^55^. Nevertheless, because any OTU may represent multiple functionally distinct bacterial strains^44^, our study may overestimate the effective overlap of leaf and root microbiomes in *B. stricta*.

## Discussion

Our study demonstrates that several sources of variation interact in complex ways to form and maintain the plant microbiome in a short-lived perennial in its native habitat. Environmental heterogeneity can decouple host genotypes from their microbiomes, just as it alters the mapping of genotype onto other aspects of the host phenotype^45^. The distinct microbial communities in different habitats can exhibit distinct responses to host genotype, host age and year-to-year variation. Our study illustrates the importance of replicating microbiome experiments across sites and timepoints, in order to reveal genotype-by-environment interactions that are potentially even more profound than environment-independent effects of host genetic variation. This approach, although laborious and challenging, may improve our ability to find genes controlling microbiome variation, and simultaneously increase our confidence that those genes are actually important in realistic environments. Such genotype–environment interactions observed between natural habitats or agroecosystems represent the cumulative influence of a potentially huge number of environmental factors. Therefore, manipulative experiments—both in the field and in controlled environments facilitating reductionist hypothesis testing—will be necessary to further disentangle these many facets of environmental complexity and learn how each affects plant microbiome composition and function^21^. Employment of additional tools including GWAS, RNAseq, metagenomics and experimental reinoculations will aid this effort. A better understanding of how host genetic variation affects associated microbial communities will be rewarding for future efforts to incorporate microbiome biology into evolutionary ecology and agricultural science.

## Methods

### Field experiment and sample collection

We collected seeds from eight to ten individual *B. stricta* plants from each of the five wild *B. stricta* populations shown in Fig. 1a, for a total of 48 accessions. These 48 genetic ‘lines' were propagated by self-fertilization in the greenhouse for at least one generation to minimize maternal environmental effects. For our analyses these lines were grouped back into five ‘genotypes' corresponding to the populations from which their ancestors were collected (Fig. 1a), because in *B. stricta* genetic variation exists predominantly among, rather than within, populations^31^.

The same batch of seeds was used for both the 2008 and 2009 cohorts. Seeds of self-full siblings of each genetic line were placed on moistened filter paper in petri dishes, stratified in the dark at 4 °C for 1 week, and then germinated in growth chamber conditions (22 °C and 11-hour days). In the Duke University greenhouses (600–2,000 μmol s^−1^ cm^−2^ photosynthetically active radiation; 65–70 °F daytime; 55–60 °F nighttime; 37–52% relative humidity), 1-week-old seedlings were transplanted into 49-ml Conetainers (Stuewe & Sons Inc., Tangent, OR, USA) filled with Fafard 4P potting soil (Conrad Fafard Inc., Agawam, MA, USA) on the bottom ∼75% and with Metromix 200 potting soil (Sun Gro Horticulture Inc., Vancouver, BC, Canada) on the top ∼25% of each pot. In October 2008 and October 2009, after ∼6–8 weeks of growth, plants were flown to Idaho and transplanted directly into the natural vegetation in each of five field sites (holes were made with a dibbler, so that no natural soil was displaced). One individual of each genotype was included in each randomized block of 48, which were planted as 10-cm grids of six rows and eight columns. Six blocks (separated by ∼1 m) were planted per site each year. In total there were 2,880 plants (48 plants per block × 6 blocks × 5 sites × 2 years). Plants were watered generously during transplanting but received no additional water or other environmental manipulations throughout the duration of the experiment.

All samples were collected in August 2011 or August 2012. Because of the extreme remoteness of these sites and difficulty of the terrain, we could only visit one site per day; however, all collections were made within a single week at the end of each growing season. All plants were rosettes at the time of harvest. Because of high mortality at the Mill Creek (Mil) and Parker Meadow (Par) sites, data from these gardens were excluded from analyses because of the resulting unbalanced design (Supplementary Table 1). At the Jackass Meadow (Jam), Mahogany Valley (Mah), and Silver Creek (Sil) sites, one individual per genotype was haphazardly selected from each experimental block in each year (Fig. 1b). In some cases, plants were too small to provide sufficient tissue from both organs, and so two different plants of the same genotype in the same block were sampled—thus, the final data set contained only 237 individuals with both leaf and root microbiome data, despite having 322 leaf and 320 root samples in total. For the same reason, samples are not perfectly distributed among blocks but blocks were sampled as evenly as possible. Leaves were collected with flame-sterilized forceps directly into autoclaved Eppendorf tubes and frozen immediately on dry ice. Roots were collected with flame-sterilized forceps into autoclaved conical 15 ml Falcon tubes on wet ice and brought back to base camp in Salmon, ID, USA, where they were rinsed and cleaned of all visible soil using distilled water and flame-sterilized utensils, and finally packed into clean autoclaved Eppendorf tubes and frozen on dry ice. We removed rhizosphere soil from roots but did not separate the epiphytic and endophytic components of either leaf or root samples; thus, DNA was extracted from microbes both on and inside leaves and roots. Simultaneously, we collected 3–7 bulk soil samples^6^ and 5–7 endogenous (that is, wild growing) control plants per site. All frozen samples were kept frozen on site at −20 °C for up to 1 week until they were shipped on dry ice to the University of North Carolina at Chapel Hill, frozen at −80 °C, and subsequently lyophilized. In summer 2013 we returned to these field sites (excepting Mill Creek for logistical reasons) and collected soil samples for chemical analysis. We collected two pints of soil from twelve spots around each site, separated by a few metres. Soil samples were analysed using established methods by the Texas A&M University AgriLife Extension Service Soil, Water, and Forage Testing Laboratory.

### Generation and processing of 16S rRNA amplicon sequences

DNA was extracted using the MoBio PowerSoil DNA Isolation Kit (MoBio Laboratories, Carlsbad, CA, USA) and DNA samples were randomized across plates. We amplified variable region V4 of the 16S small subunit ribosomal gene. The forward primer consisted of the Illumina adapter sequence (5′- AATGATACGGCGACCACCGAGATCTACAC -3′) attached to the Read 1 sequencing primer-binding site (5′- TCTTTCCCTACA -3′) followed by 0–5 random bases then established primer 515F (5′- GTGCCAGCMGCCGCGGTAA -3′). The reverse primer was made by combining the Illumina adapter sequence (5′- CAAGCAGAAGACGGCATACGAGAT -3′) with a 12-bp barcode, the Read 2 sequencing primer-binding site (5′- GTGACTGGAGTTCAGACGTGTGCTCTTCCGATCT -3′), a stretch of 0–3 random nucleotides to increase sequence complexity, and the established amplification primer 806R (5′- GGACTACHVGGGTWTCTAAT -3′)^56^. PNA PCR clamps were used to reduce host organelle contamination^57^. Each PCR reaction was prepared with 10 μl 5 Prime Hot Master Mix (Hilden, Germany), 0.5 μl forward primer (10 μM), 0.5 μl reverse primer (10 μM), 1 μl template DNA (∼10 ng μl^−1^), 1 μl bovine serum albumin (10 mg ml^−1^), and 12 μl PCR-grade water. PCR amplifications (performed in triplicate for each sample) consisted of a 3 min denaturation at 94 °C; 30 cycles of 45 s at 94°C, 60 s at 50 °C and 90 s at 72 °C; and 10 min at 72 °C. Samples were cleaned using AmPureXP magnetic beads (Agencourt, Beverly, MA, USA), pooled in equimolar concentrations and sequenced at 96-plex at the Joint Genome Institute (Walnut Creek, CA, USA) using 2 × 250 bp MiSeq v2 sequencing (Illumina Inc., San Diego, CA, USA), resulting in 75,270±39,601 high-quality reads per sample (mean±1 s.d.). After trimming primer sequences, the amplicon sequences were processed using FLASH^58^ and UPARSE^59^ software. Sequences were grouped into OTUs based on 97% or greater identity, and assigned taxonomy by comparison to the Greengenes^60^ database (May 2013 version) using the QIIME implementation of the RDP classifier^61^,^62^. OTU representative sequences were aligned and filtered using QIIME 1.7.0, and a rooted phylogenetic tree was generated using the midpoint method^61^. OTUs with no kingdom-level classification or matching chloroplast, mitochondrial or Viridiplantae sequences were then removed from the data set. At this point, we omitted low-coverage samples (<800 usable reads) from the data set, resulting in 322 leaf and 320 root samples proceeding to downstream analysis. This data set included 16 leaf and 10 root samples from endogenous control plants, which were used for UniFrac calculations and PCoA (below) but then excluded from analyses of experimental factors. As a final filter, OTUs were classified as ‘non-reproducible' and removed from the data set unless they were observed at least 25 times in at least five samples^16^. A total of 3,718 reproducible, measurable OTUs comprised the final data set. Finally, the raw counts of each OTU were divided by estimated 16S gene copy number^34^ and then rounded up to integer values before all downstream analysis.

### Statistical analysis

We analysed all the data and made all figures in R version 3.2.3 using the Phyloseq and ggplot2 packages^63^,^64^. All R code used to generate results and figures are available along with the raw data in a Dryad repository (doi:10.5061/dryad.g60r3). We analysed the leaf and root data separately except when directly comparing the two organs within a single plant (see below). We controlled for technical noise (variation attributable to sequencing depth or batch effects) by including MiSeq run as a random effect and total number of observations as a covariate in statistical models. We assessed statistical significance at *α*=0.05 throughout and whenever necessary, we adjusted *P* values for multiple comparisons using the sequential Bonferroni correction.

### Analysis of OTU sharing between leaves and roots

For these analyses only, we used OTUs binned at a more stringent 99% sequence similarity threshold. To assess overlap between leaf and root communities we restricted analysis to the subset of plants for which we had both leaf and root data (*N*=237). Because these analyses only compared within-plant leaf and root communities and therefore did not require a balanced experimental design, we included endogenous and experimental plants from all five field sites, including those where low survival prohibited other statistical analyses (Supplementary Table 1). For each of these plants, we determined the ‘shared set' of OTUs that were observed in both the leaf and the corresponding root. We recorded the relative abundance of each of these OTUs separately in the leaf and root, generating a large table in which each OTU was represented once for each plant in which it was found (that is, up to 237 times) by paired leaf and root relative abundances. We applied a LMM to these paired data to test for a correlation between relative abundance in the two organs: ln(relative abundance in the leaf)=ln(relative abundance in the root)+OTU_ID+Plant_ID, where OTU_ID and Plant_ID were categorical random variables that allowed the intercept to vary for each OTU and each plant.

We then asked what proportion of the leaf microbiome was shared with the root microbiome, and vice versa. For each plant, we summed the relative abundances of the OTUs in that plant's ‘shared set' for each leaf sample; then, we did the same for each plant's corresponding root sample. To summarize the experiment-wide overlap between leaf and root OTUs, we pooled the shared OTUs of all 237 plants and calculated the relative abundance of each in leaves and roots.

### Microbiome data normalization

For downstream applications that assume homoscedasticity or can be influenced by unequal variances (PCoA and LMMs) we normalized OTU counts using the variance-stabilizing transformation^32^. For downstream NBMs, we used raw (un-normalized) OTU counts^32^,^33^.

### Principal coordinates analysis and α-diversity calculations

Unconstrained PCoA was performed using the function capscale() in the vegan package^65^. The input to capscale() was the matrix of UniFrac distances^35^ generated from normalized OTU counts. To assess variation in both relative abundance and presence/absence, we analysed both weighted and unweighted UniFrac distances^35^. Sample scores on the PCoA axes were saved and used as response variables in separate LMMs (below). For each sample, Chao1 richness and Shannon diversity were calculated using the estimate_richness() function of the Phyloseq package^63^.

### Linear mixed models

We fit all LMMs using the lme4 package^66^. Predictor variables in these models included the fixed effects Site+Genotype+Genotype x Site+Age+Age x Site+Year+Year × Site+ln(Total observations), and the random effects Line+Block+MiSeq run. Lines were nested in Genotypes, and Blocks nested in Sites. The ‘Total observations' covariate is the total number of usable (non-host) reads from each sample (after adjustment for copy number variation). We assessed statistical significance of fixed predictors using Type III ANOVA with Satterthwaite's approximation of denominator degrees of freedom in the package lmerTest^67^, and of random effects using likelihood ratio tests. We used this model to predict community descriptors that were continuous and approximately normally distributed: both α-diversity metrics (Shannon entropy and Chao1 estimated richness, calculated using the Phyloseq package^63^) and β-diversity metrics (top three PCoA axes of weighted and unweighted UniFrac distance matrices, as described above).

### Prediction of abundance of individual bacterial taxa

We also modelled abundance of individual OTUs, families, orders, classes and phyla. We excluded the rarest OTUs from these analyses, only analysing OTUs contributing at least 10% of the mean OTU abundance; that is, we kept only leaf OTUs that were observed at least 169 times (10% of 1,693, the mean leaf OTU count) and root OTUs that were observed at least 310 times (10% of 3,109, the mean root OTU count). This process reduced the leaf data set to 1,016 OTUs and the root data set to 2,666 OTUs, but retained >98% of all observations in both organs. For analyses of higher-level taxa, we condensed the ‘unfiltered' set of OTUs (that is, before thresholding to remove ‘non-reproducible' OTUs) into matrices of family, order, class and phylum counts using the tax_glom() function in the Phyloseq package^63^. We again excluded taxa whose total abundances were less than 10% of the mean abundance (see above).

At all five taxonomic levels, we modelled raw (non-normalized) taxon counts using negative binomial generalized linear models, implemented in the package DESeq2 (ref. 32). The NBMs included the fixed effects Site+Genotype+Age+Year. We asked whether each of these main effects predicted the abundance of each taxon by performing a likelihood ratio test comparing the deviance of this full model to the deviance of the reduced model, with one term excluded; for example, to test for any change in abundance attributed to Site, we compared the full model above to a reduced NBM with only the fixed effects Genotype+Age+Year. To ask whether the interaction of main effects with Site predicted taxon abundance, we used likelihood ratio tests to compare the deviance of a model including the interaction term to the deviance of the base model described above, with all main effects but no interaction. For example, to test for genotype-by-site interactions we compared the NBM with fixed effects Genotype+Site+Age+Year+Genotype × Site to the NBM with fixed effects Genotype+Site+Age+Year. *P* values resulting from these likelihood ratio tests were adjusted for multiple comparisons using the Benjamini–Hochberg false discovery rate^68^.

To obtain estimates of effect sizes, for each taxon we estimated log_2_ fold change between pairs of experimental groups (for example, between two field sites or two genotypes) and assessed statistical significance of each contrast using a Wald test at *P*<0.05 after correction for multiple comparisons using the Benjamini–Hochberg false discovery rate^68^ (for example, for the Site term, we tested differential abundance between a pair of field sites for all 1,016 leaf OTUs). Because multiple Wald tests were conducted for each term in the model which had >2 levels (that is, genotype, site, age and interaction terms), we further adjusted these *P* values to correct for these multiple pairwise comparisons, again using the Benjamini–Hochberg method. After correction, nonsignificant contrasts were considered to have an effect size (log_2_ fold change) of zero.

### Heritability of individual bacterial taxa

We estimated broad-sense heritability (H^2^) for bacterial taxa in each site, excluding the rarest OTUs as described above. To make the data suitable for variance-partitioning using ANOVA, taxon counts were normalized using a variance-stabilizing transformation implemented in the R package DESeq2 (ref. 32). To estimate heritability across all sites, we fit the random effects linear model Site+Genotype+Genotype*Site+Age+Age*Site+Year+Year*Site+Line+Block+MiSeq run+ln(Total observations), and calculated H^2^ as the sum of the Genotype, Genotype*Site and Line variance components divided by the sum of all variance components (including residual variance). To estimate heritability within each site, the transformed counts were subsetted by field site and modelled using the random effects linear model Genotype+Age+Year+Line+Block+MiSeq run+ln(Total observations). We estimated H^2^ as the sum of the Genotype and Line variance components divided by the sum of all variance components (including residual variance).

### Data availability

Raw reads sequencing reads have been deposited at the European Nucleotide Archive, with study number ‘PRJEB10570'. All R code used to generate results and figures has been deposited along with the processed data in a Dryad repository (doi: 10.5061/dryad.g60r3)^69^. The authors declare that all other data that support the findings of this study are included in the manuscript and its supplementary files or are available from the corresponding author upon request.

## Additional information

**How to cite this article:** Wagner, M. R. *et al*. Host genotype and age shape the leaf and root microbiomes of a wild perennial plant. *Nat. Commun.* 7:12151 doi: 10.1038/ncomms12151 (2016).

## Supplementary Material

Supplementary Information Supplementary Figures 1 - 14, Supplementary Tables 1 - 7, Supplementary Notes 1 and 2 and Supplementary References

Supplementary Data 1 Database of all bacterial taxa predicted by experimental factors in negative binomial models. Dataset is stored as a tab-delimited text file. Each row represents one bacterial Taxon tested in either leaves or roots (column "Organ"). Column "Level" refers to the taxonomic level of organization at which the analysis was performed. Column "Term" refers to the experimental factor that predicted abundance of the Taxon in the negative binomial model ("GxS"=genotype-by-site interactions; "AxS"=age-by-site interactions; "Year"=year of harvest; "YxS" = year-by-site interactions). Only statistically significant results from the likelihood ratio test are included in this database (after controlling the false discovery rate at alpha=0.05 using the Benjamini-Hochberg method; adjusted P-values from the likelihood ratio test are listed in the column "padj"). The remaining columns show the relative abundance of the taxon in various subsets of the dataset, coded as follows: "3sites" = all samples from Jam, Mah, Sil combined; "5sites" = samples from all five field sites combined; "Jam", "Mah", "Mil", "Par", "Sil" = samples from each of these sites (note that data from "Mil" and "Par" were not used in these models due to unbalanced design resulting from low survival; see Supplementary Table 1); "Age2", "Age3", "Age4" = samples in each age group, only from the 3 main field sites used in this model (i.e., "Jam", "Mah", and "Sil"); "endog" = samples from endogenous plants, only from the 3 main field sites. The endogenous plants also were not included in this model. Note that relative abundances may not sum to 1 because not all taxa are represented in this table (some were not predicted by any term in the negative binomial models). The provided relative abundances are organ-specific; for example, "RelAbund_Jam" is the relative abundance of a taxon either in leaves OR roots (depending on the value in the 'Organ' column) sampled from Jackass Meadow.

Supplementary Data 2 Database of putatively unnatural OTUs persisting from potting-soil inoculum. Dataset is stored as a tab-delimited text file. These 29 OTUs were identified as being present in experimentally transplanted individuals but not in endogenous plants or wild bulk soil samples, and therefore potentially endemic to the greenhouse/potting soil. Reference sequences and taxonomic designations are provided. The remaining columns show the relative abundance of the taxon in various subsets of the dataset, coded as described for Supplementary Dataset 1.

## Figures and Tables

**Table 1:** Experimental factors predicting α-diversity of leaf- and root- associated bacterial communities.

	**Leaf**		**Root**	
	**Shannon**	**Chao1**	**Shannon**	**Chao1**
*R*^2^	0.56	0.63	0.68	0.72
Site	*F*_2,48_=44.1	*F*_2,49_=51	*F*_2,41_=140	*F*_2,34_=141
	***P*****=1.4e**−**11**	***P*****=2.3e−12**	***P*****<3e−16**	***P*****<3e−16**
Geno.	*F*_4,263_=1.62	*F*_4,258_=4.68	*F*_4,28_=0.648	*F*_4,261_=0.344
	*P*=0.17	***P*****=0.0023**	*P*=1	*P*=1
Geno. × Site	*F*_8,261_=2.33	*F*_8,256_=0.903	*F*_8,260_=1.73	*F*_8,259_=0.94
	***P*****=0.04**	*P*=0.51	*P*=0.18	*P*=0.48
Age	*F*_2,61_=0.652	*F*_2,64_=1.11	*F*_2,67_=2.91	*F*_2,57_=6.08
	*P*=0.67	*P*=0.67	*P*=0.062	***P*****=0.0081**
Age × Site	*F*_4,59_=1.63	*F*_4,63_=1.48	*F*_4,65_=0.724	*F*_4,56_=7.85
	*P*=0.36	*P*=0.36	*P*=0.58	***P*****=8.6e−05**
Year	*F*_1,73_=7.36	*F*_1,66_=8.37	*F*_1,54_=6.73	*F*_1,72_=12.3
	***P*****=0.01**	***P*****=0.01**	***P*****=0.012**	***P*****=0.0016**
Year × Site	*F*_2,79_=0.741	*F*_2,71_=0.193	*F*_2,88_=0.153	*F*_2,73_=5.94
	*P*=0.96	*P*=0.96	*P*=0.86	***P*****=0.0082**
Block	*χ*^2^_1_=1.48	*χ*^2^_1_=6.27	*χ*^2^_1_=1.13	*χ*^2^_1_=2.08
	*P*=0.22	***P*****=0.025**	*P*=0.3	*P*=0.3
Line	*χ*^2^_1_=7.96e-13	*χ*^2^_1_=0	*χ*^2^_1_=0.0128	*χ*^2^_1_=0
	*P*=1	*P*=1	*P*=1	*P*=1
Obs	*F*_1,273_=3.08	*F*_1,267_=132	*F*_1,180_=0.4	*F*_1,137_=104
	*P*=0.081	***P*****<3e−16**	*P*=0.53	***P*****<3e−16**
MiSeq run	*χ*^2^_1_=0.754	*χ*^2^_1_=0.649	*χ*^2^_1_=1.79	*χ*^2^_1_=0.123
	*P*=0.77	*P*=0.77	*P*=0.36	*P*=0.73

ANOVA, analysis of variance.

Statistics describe linear random-intercept models of Shannon diversity and Chao1 richness in leaves and roots. ‘Obs', total number of observations (log-transformed). All *P* values were corrected for multiple comparisons using the sequential Bonferroni correction. Significance was assessed using Type III ANOVA with F tests for fixed effects and likelihood ratio tests for random effects. Bold values indicate statistically significant results after correction for multiple comparisons, P<0.05.

**Table 2:** Experimental factors predicting β-diversity of leaf- and root- associated bacterial communities.

	**Leaf**			**Root**		
	**PCo1**	**PCo2**	**PCo3**	**PCo1**	**PCo2**	**PCo3**
*R*^2^	0.84	0.63	0.64	0.89	0.76	0.44
Site	*F*_2,55_=232	*F*_2,48_=39	*F*_2,46_=73.3	*F*_2,35_=457	*F*_2,32_=70.9	*F*_2,38_=20.9
	***P*****<3e−16**	***P*****=8.8e−11**	***P*****=1.1e−14**	***P*****<3e−16**	***P*****=4.9e−12**	***P*****=8.6e−07**
Geno.	*F*_4,12_=7.65	*F*_4,261_=3.82	*F*_4,13_=4.08	*F*_4,25_=0.224	*F*_4,17_=0.234	*F*_4,28_=0.118
	***P*****=0.0088**	***P*****=0.0098**	***P*****=0.026**	*P*=1	*P*=1	*P*=1
Geno. × Site	*F*_8,257_=4.53	*F*_8,259_=1.89	*F*_8,258_=1.27	*F*_8,255_=1.04	*F*_8,255_=0.312	*F*_8,260_=0.857
	***P*****=0.00011**	*P*=0.12	*P*=0.26	*P*=1	*P*=1	*P*=1
Age	*F*_2,65_=0.789	*F*_2,63_=3.34	*F*_2,63_=0.734	*F*_2,55_=0.43	*F*_2,49_=61.2	*F*_2,64_=13.4
	*P*=0.92	*P*=0.13	*P*=0.92	*P*=0.65	***P*****=1.5e−13**	***P*****=2.8e−05**
Age × Site	*F*_4,62_=1.55	*F*_4,61_=0.229	*F*_4,61_=1.26	*F*_4,55_=2.31	*F*_4,48_=10.8	*F*_4,62_=2.87
	*P*=0.6	*P*=0.92	*P*=0.6	*P*=0.07	***P*****=7.7e**^**−06**^	*P*=0.06
Year	*F*_1,59_=5.88	*F*_1,69_=0.009	*F*_1,41_=3.88	*F*_1,61_=18.4	*F*_1,60_=7.65	*F*_1,61_=25.2
	*P*=0.055	*P*=0.93	*P*=0.11	***P*****=0.00013**	***P*****=0.0076**	***P*****=1.4e**−**05**
Year × Site	*F*_2,97_=0.511	*F*_2,73_=1.27	*F*_2,68_=0.975	*F*_2,65_=2.54	*F*_2,66_=6.94	*F*_2,93_=0.178
	*P*=0.86	*P*=0.86	*P*=0.86	*P*=0.17	***P*****=0.0055**	*P*=0.84
Block	*χ*^2^_1_=0.06	*χ*^2^_1_=4.54	*χ*^2^_1_=3.59	*χ*^2^_1_=5.49	*χ*^2^_1_=1.26	*χ*^2^_1_=0.09
	*P*=0.81	*P*=0.099	*P*=0.12	*P*=0.057	*P*=0.52	*P*=0.76
Line	*χ*^2^_1_=0.16	*χ*^2^_1_=0	*χ*^2^_1_=1.3e−05	*χ*^2^_1_=1.00	*χ*^2^_1_=0.28	*χ*^2^_1_=1.53
	*P*=1	*P*=1	*P*=1	*P*=0.65	*P*=0.65	*P*=0.65
Obs	*F*_1,279_=17.3	*F*_1,273_=156	*F*_1,239_=24.7	*F*_1,177_=8.84	*F*_1,205_=3.2	*F*_1,127_=19.1
	***P*****=4.2e−05**	***P*****<3e−16**	***P*****=2.6e−06**	***P*****=0.0067**	*P*=0.075	***P*****=7.5e−05**
MiSeq run	*χ*^2^_1_=4.62	*χ*^2^_1_=1.41	*χ*^2^_1_=0.001	*χ*^2^_1_=1.2	*χ*^2^_1_=2.94	*χ*^2^_1_=0.28
	*P*=0.095	*P*=0.47	*P*=0.97	*P*=0.55	*P*=0.26	*P*=0.59

ANOVA, analysis of variance.

Statistics describe linear random-intercept models of weighted UniFrac principal coordinates in leaves and roots. ‘Geno', plant genotype; and ‘Obs', total number of observations (log-transformed). All *P* values were adjusted for multiple comparisons using the sequential Bonferroni correction. Significance was assessed using type III ANOVA with F tests for fixed effects and likelihood ratio tests for random effects. Bold values indicate statistically significant results after correction for multiple comparisons, P<0.05.
