# Supplementary material for: Host genotype and age shape the leaf and root microbiomes of a wild perennial plant
Source: Nat Commun. 2016 Jul 12;7:12151. doi: 10.1038/ncomms12151 (PMC4945892; doi:10.1038/ncomms12151)
Supplement: Supplementary Information — Supplementary Figures 1 - 14, Supplementary Tables 1 - 7, Supplementary Notes 1 and 2 and Supplementary References [file ncomms12151-s1.pdf]

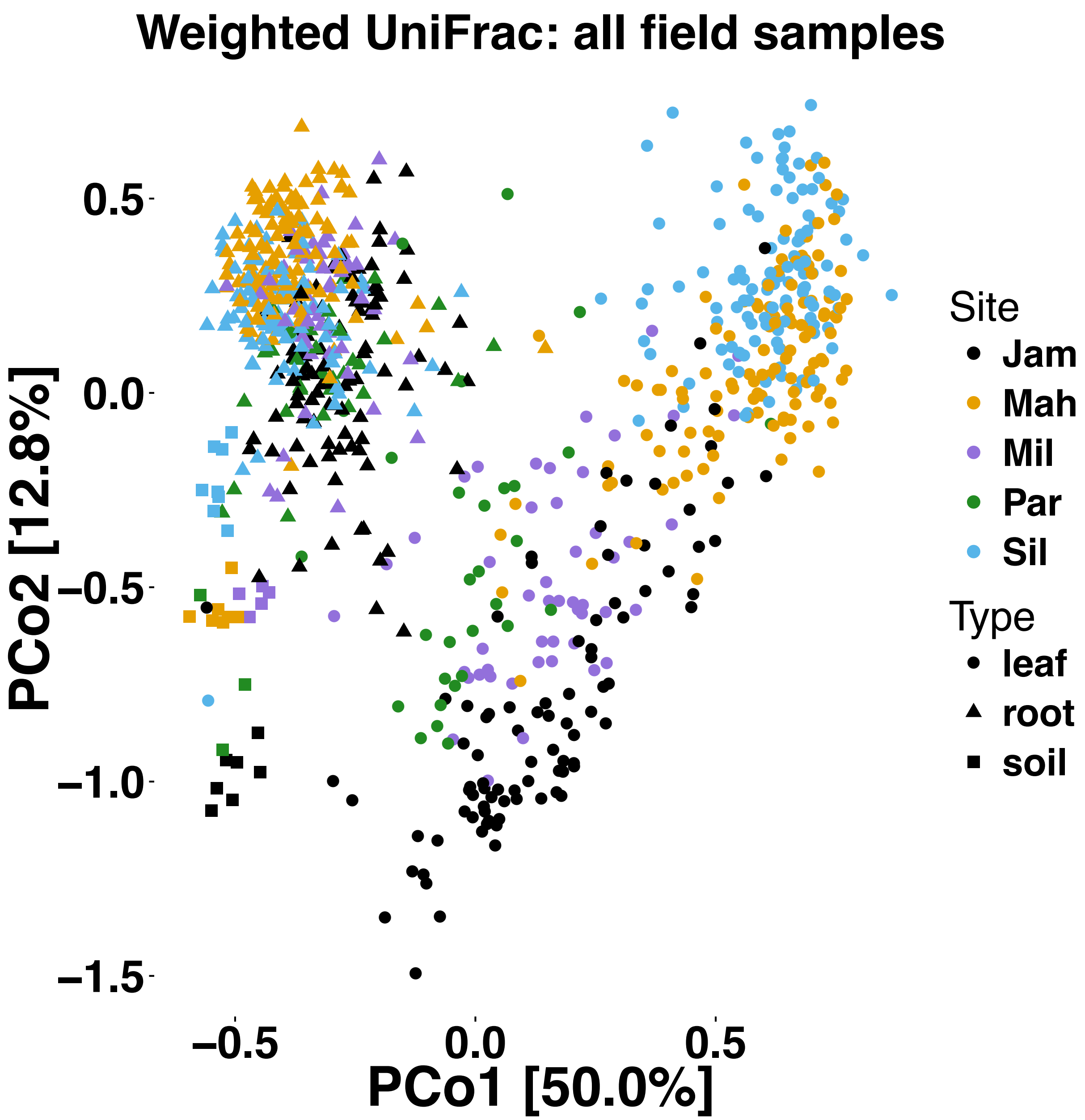

Supplementary Figure 1 | Bacterial microbiomes differ between plant organs and between habitats. Unconstrained principal coordinates analysis of weighted UniFrac distances between leaf (N=402), root (N=407), and bulk soil (N=30) samples from five natural habitats of the wild perennial mustard *Boechera stricta* reveals that these field sites harbor distinct bacterial communities. Leaf-associated and root-associated bacterial communities are dramatically different, and both are distinguished from bulk soil communities.

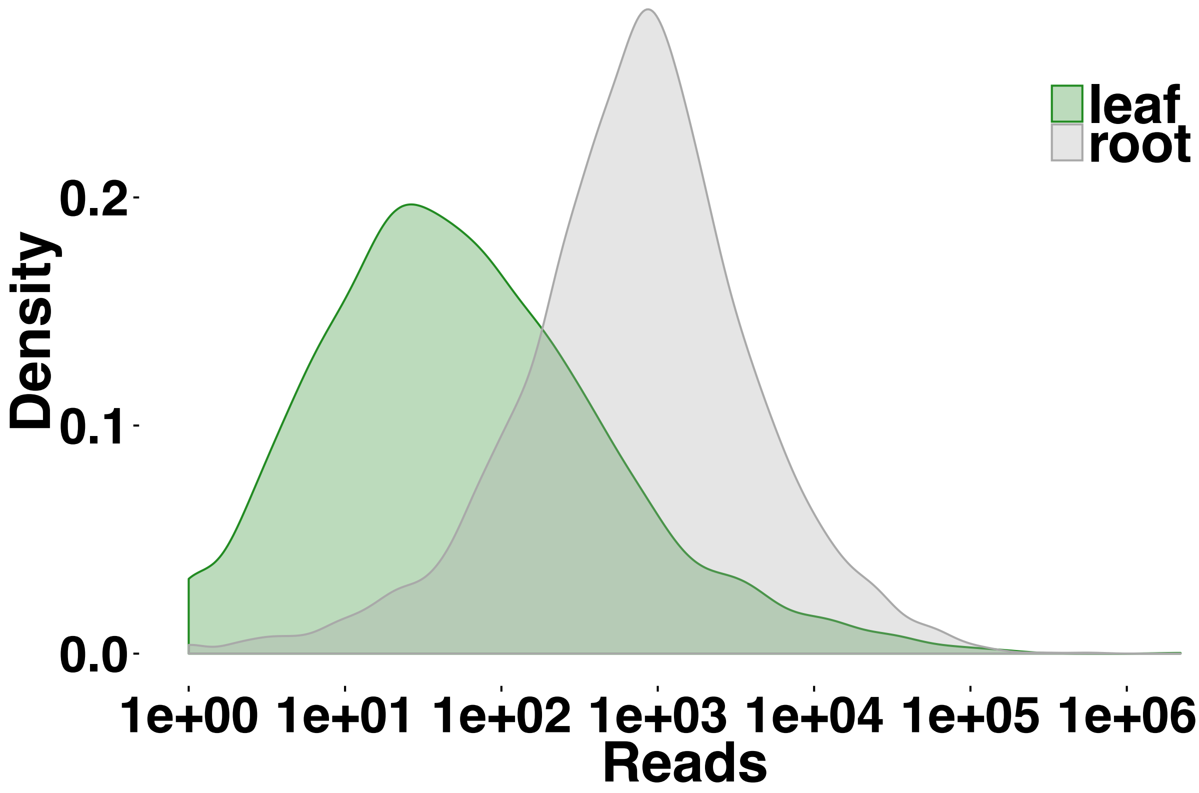

Supplementary Figure 2 | Distributions of OTU counts were especially skewed in leaves. Density plots of OTU counts in leaves and roots (note log scale on horizontal axis).

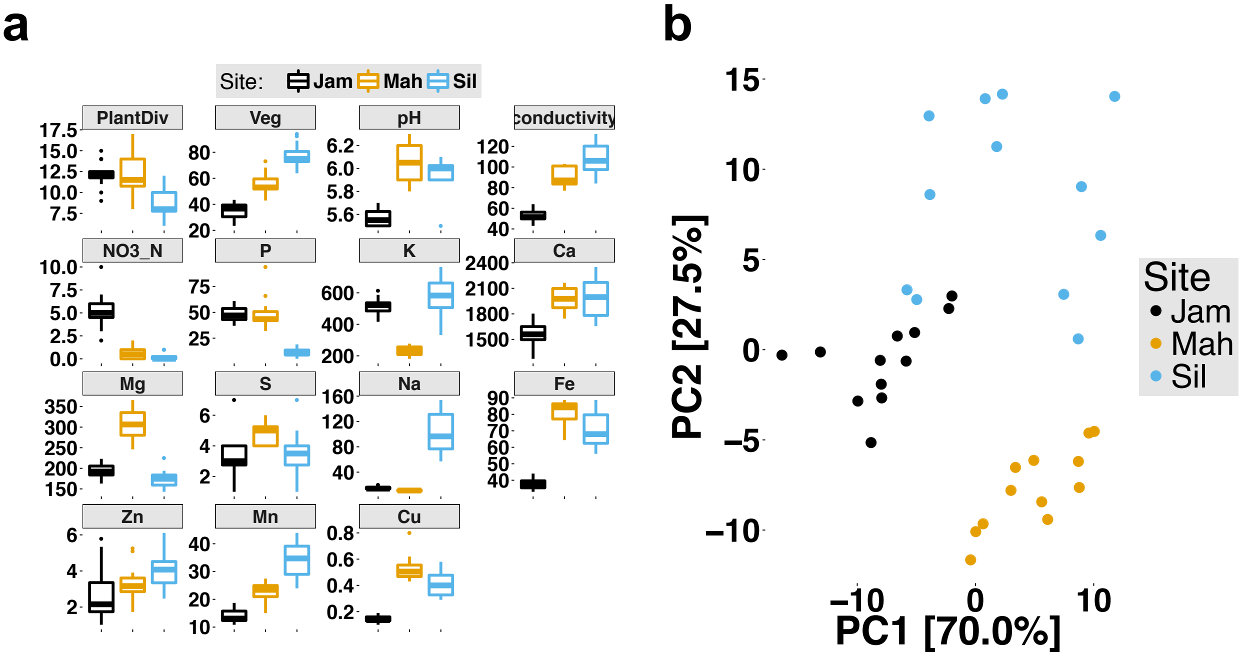

Supplementary Figure 3 | Biotic and abiotic environmental variation in three natural *Boechera stricta* habitats featured in this study. (a) Data were collected in summer 2013 from 12 half-square-meter plots spanning each common garden. "PlantDiv" = number of plant morphospecies present in each block (not counting *B. stricta*); "Veg" = percent vegetation cover of each block (estimated for each of 50 sub-blocks of area 10x10cm, then aggregated). All other variables describe chemical content of soils (units: pH = none, conductivity = umho/cm, all others = ppm). The bottom and top edges of the boxes mark the 25th and 75th percentiles (i.e., first and third quartiles). The horizontal line within the box denotes the median. Whiskers mark the range of the data excluding outliers that fell more than 1.5 times the interquartile range below the first quartile or above the third quartile (dots). **(b)** Principal components analysis of the environmental variables shown in (a).

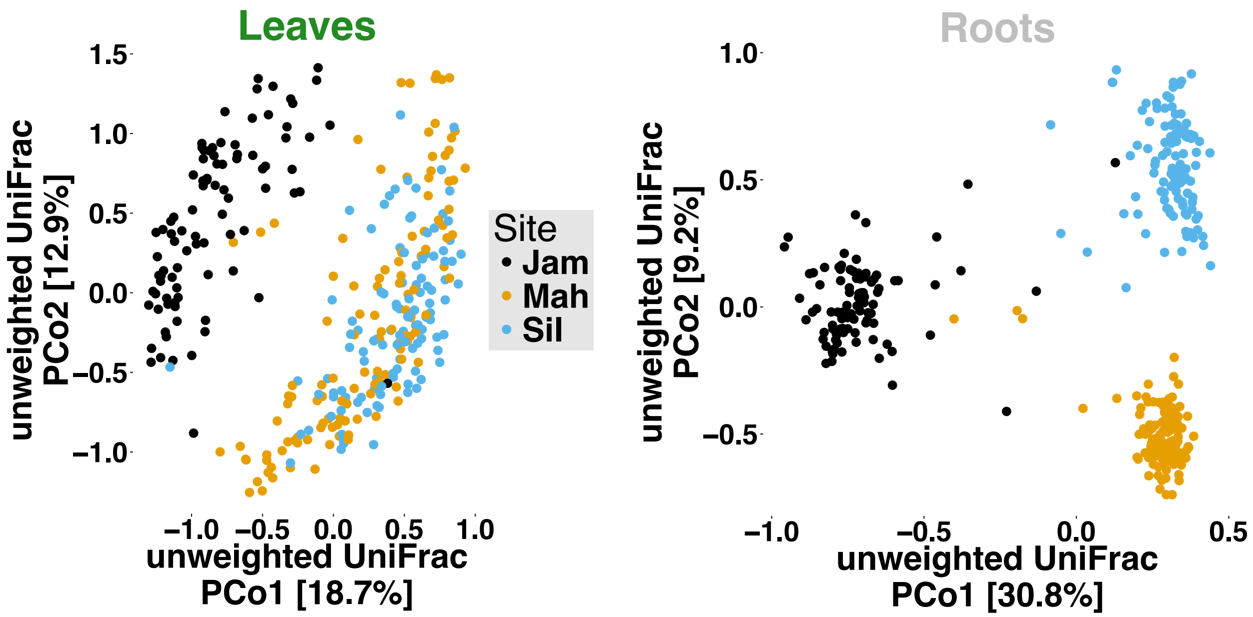

Supplementary Figure 4 | Leaf and root microbiomes differ substantially between *B. stricta* habitats. Principal coordinates analysis of unweighted UniFrac distances between samples reveals strong separation of leaf and root community composition among field sites. ANOVA, P<0.05; detailed statistics are found in Supplementary Table 5.

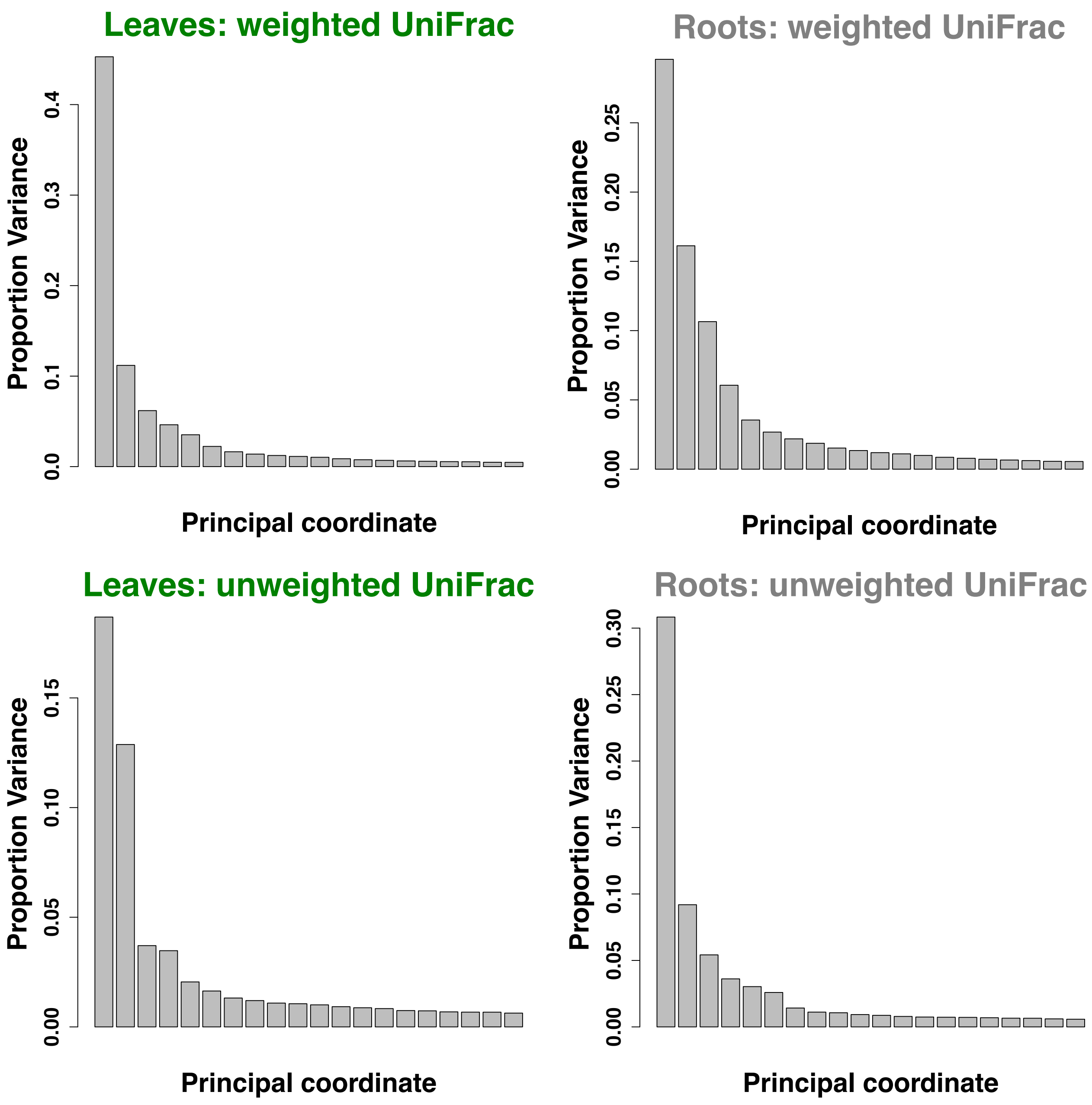

Supplementary Figure 5 | The major three PCoA axes represent >50% of the total variation in *B. stricta* microbiomes. Scree plots for PCoA of weighted UniFrac distances for leaves and roots (only top 20 axes are shown).

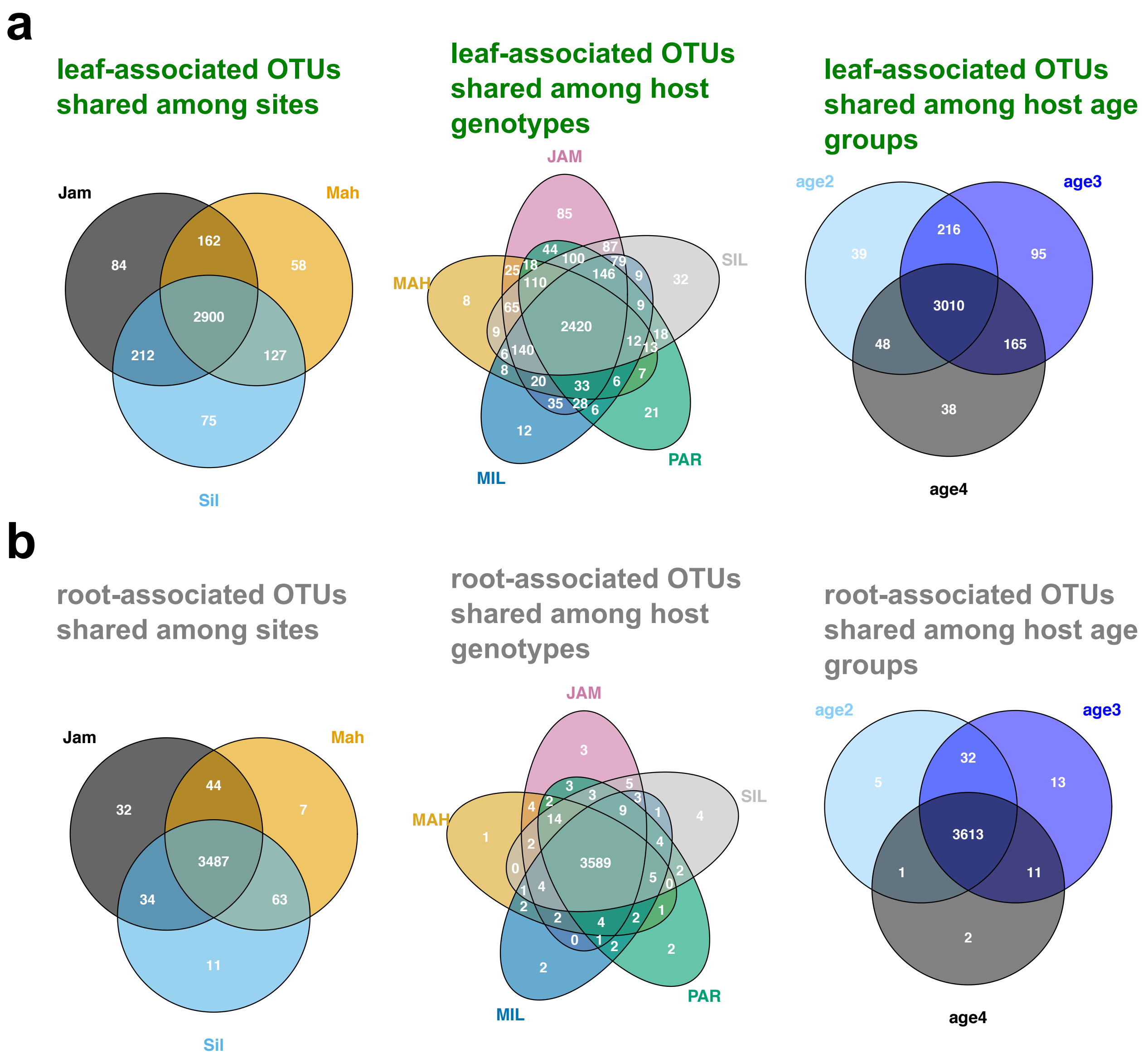

Supplementary Figure 6 l Overlap in OTU membership of plant-associated bacterial communities. A large proportion of bacterial OTUs was shared among field sites, plant genotypes, and plant age groups in both (a) leaves and (b) roots.

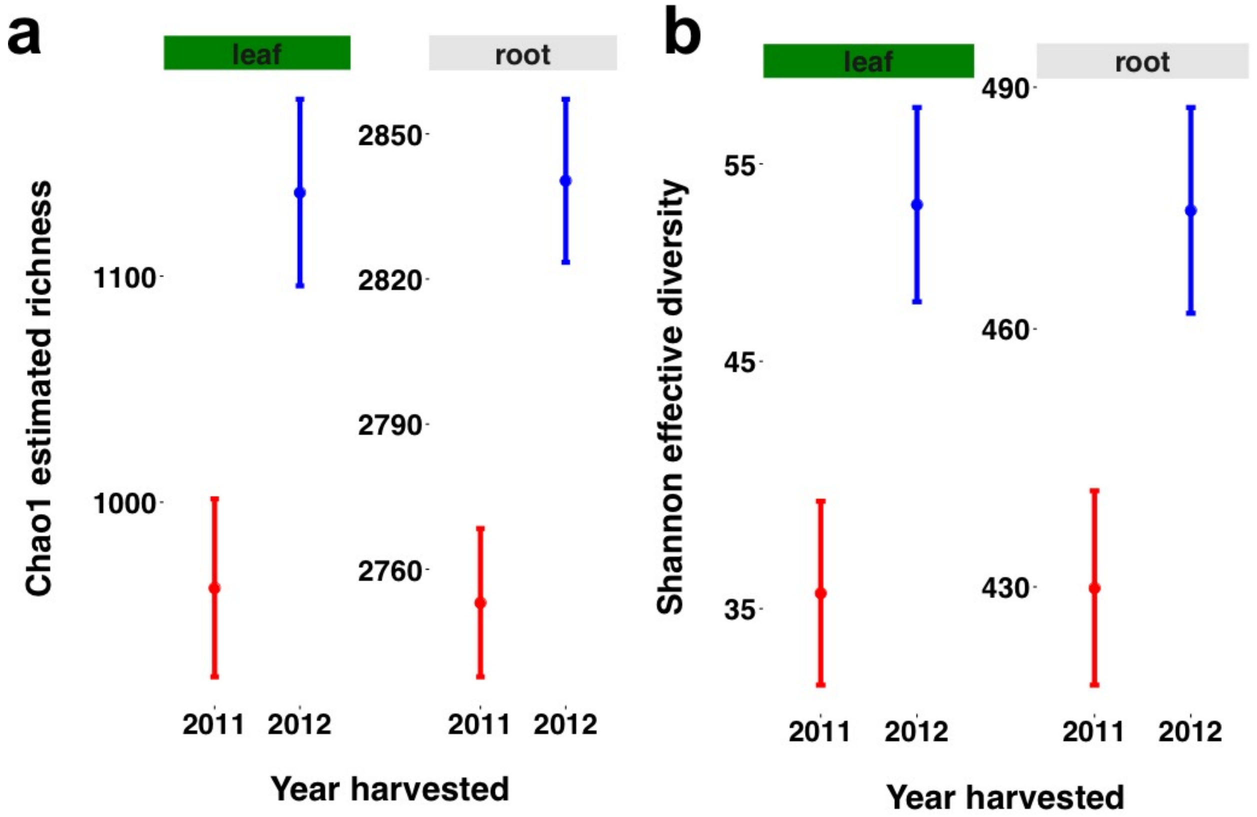

Supplementary Figure 7 | Consistent interannual variation in *B. stricta* microbiome richness. Alpha diversity increased between 2011 and 2012 in both leaves and roots. Plotted are (**a**) least squares mean Chao1 richness and **(b)** least squares mean effective Shannon diversity ($e^{\text{Shannon entropy}}$) in each year after controlling for other sources of variation. Statistics associated with these models are found in Table 1. Bars depict one standard error of the mean.

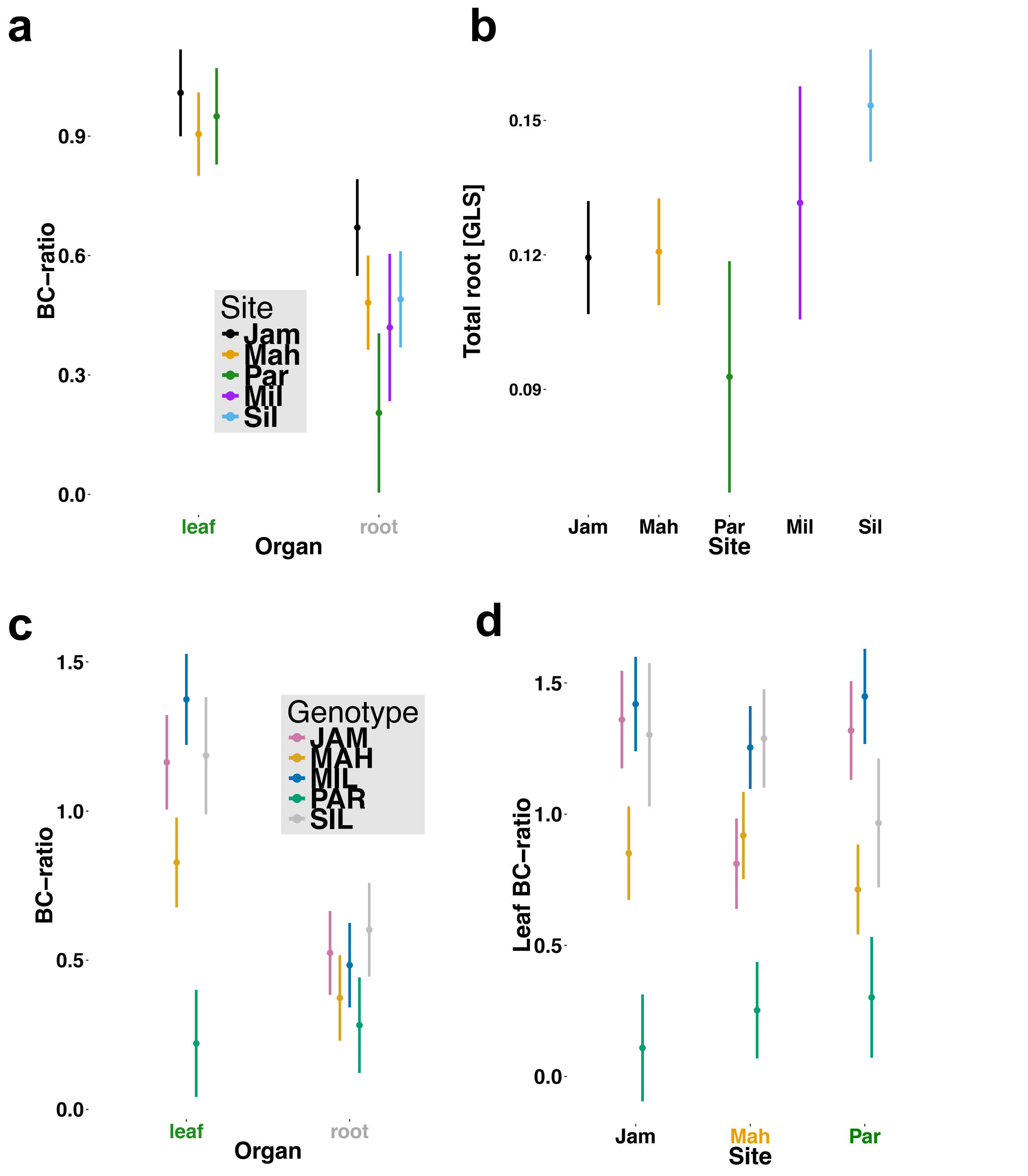

Supplementary Figure 8 I Genotype, site, and genotype-by-site interactions shape glucosinolate (GLS) content. Least-squares mean values (plus or minus one standard error of the mean) are plotted for Total glucosinolate concentration and for BC-ratio, a measurement of glucosinolate quality. (a) In this study, BC-ratio was plastic among sites for roots but not leaves; (b) total root glucosinolate concentration was plastic among sites; (c) Genotype controls BC-ratio in leaves but not roots; (d) genotype effects on leaf BC-ratio are site-dependent.

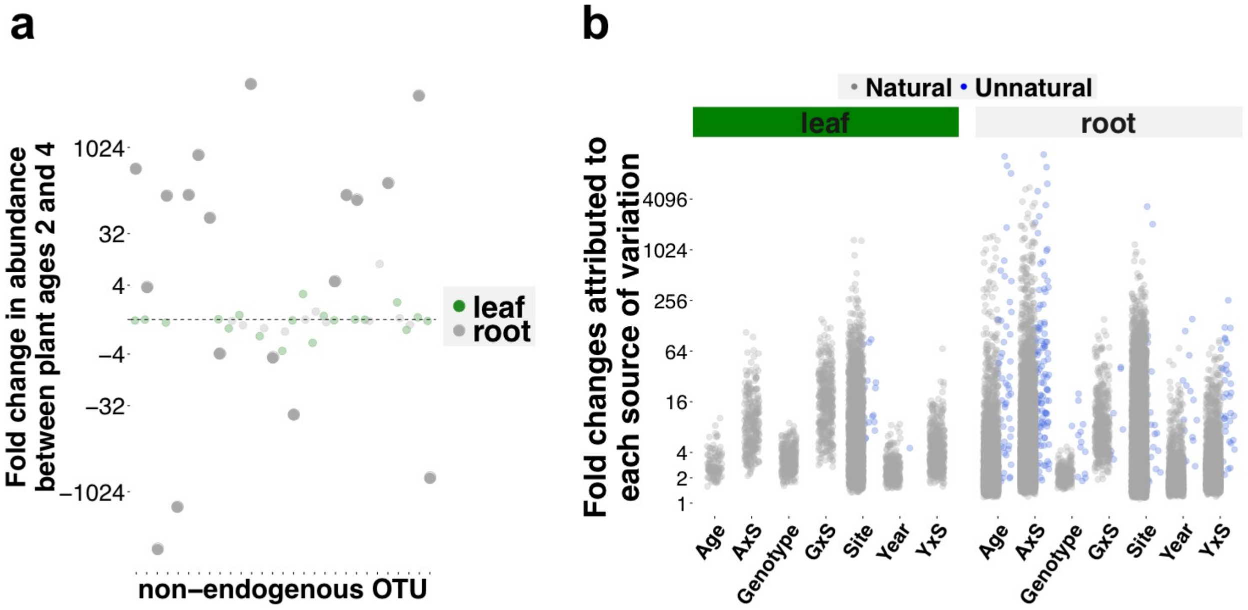

Supplementary Figure 9 | Putatively unnatural OTUs are otherwise unremarkable. Twenty-nine OTUs were identified as being present in roots of experimentally transplanted individuals but not in any endogenous plant or wild bulk soil sample. **(a)** The fold change in abundance between 2-year-old and 4-year-old plants (estimated using NBMs as described in the main text) is plotted for the 29 "unnatural" OTUs in leaves and roots. Small, faded points indicate changes that were not significantly different from zero. Only 6 of these OTUs show the expected decrease in abundance with time since transplant, suggesting that some may in fact have been rare natural bacteria that were not observed in the relatively small number of wild samples, simply by chance; nevertheless we excluded them from other analyses as a conservative precaution. **(b)** Magnitudes of fold changes in abundance due to each source of variation are plotted for "natural" and "unnatural" OTUs. The sensitivity of the 29 unnatural OTUs to host genotype, age, and site is comparable to that shown by the natural OTUs. Note that whereas panel (a) only shows fold changes between 2- and 4-year old plants, here fold changes between 2- and 3-year old plants and between 3- and 4-year old plants are also shown.

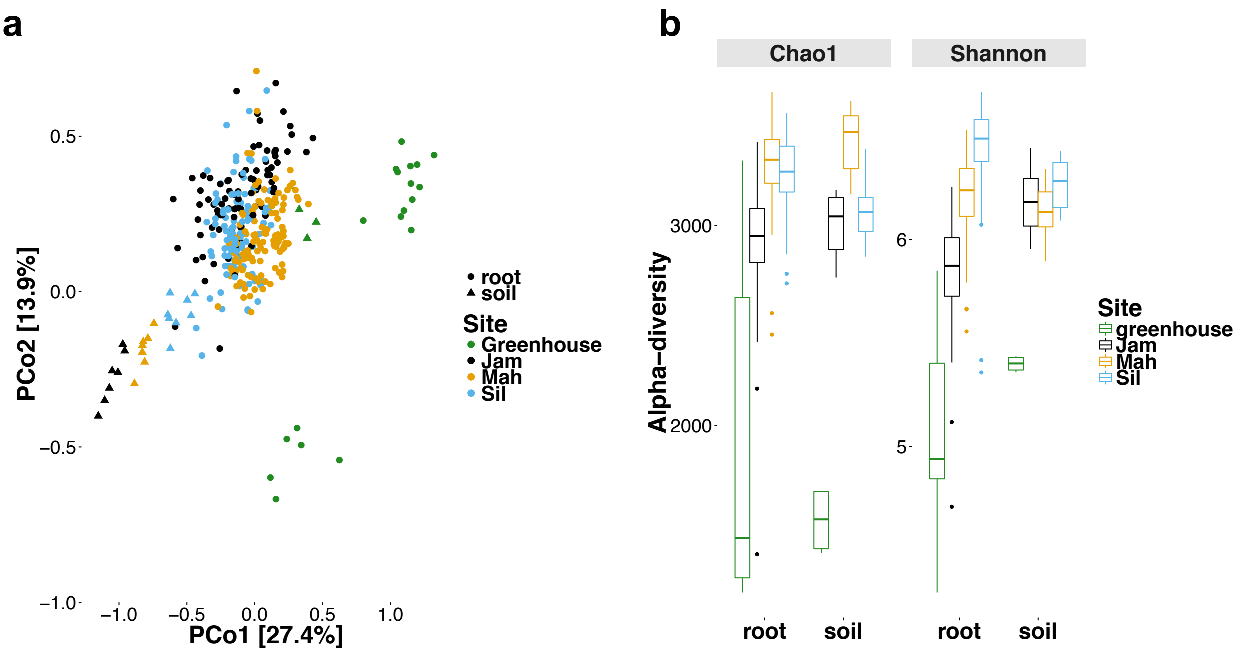

Supplementary Figure 10 | The root microbiomes of greenhouse-grown plants and potting soils differ strongly from those in natural environments. (a) Principal coordinates analysis of weighted UniFrac distances reveals that despite extensive differences among field sites in bacterial community diversity and composition (see main manuscript Tables 1-2, Figs. 2-3), root communities in the three field sites are much more similar to each other than they are to root communities of plants grown in potting soil in the greenhouse. **(b)** Alpha diversity was much lower in potting soil (and in *B. stricta* roots grown in potting soil) than in bulk soils or roots at any of the field sites.

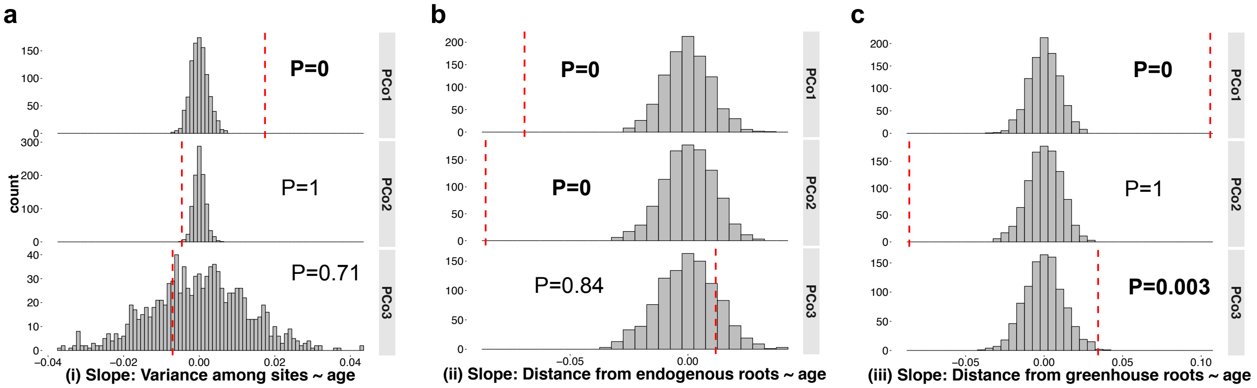

Supplementary Figure 11 I Not all root community changes driven by plant age are consistent with the hypothesis that age effects are only due to post-transplant succession. Histograms show the distribution of "succession parameters" calculated for simulated datasets with plant age permuted (i.e., under the null hypothesis of no ongoing turnover); vertical dashed lines show the true/observed value of each parameter. Small P-values indicate that the data are consistent with the hypothesis that community turnover after transplant fully explains the effect of host age on root bacterial microbiomes. **(a)** The variance in mean PCo1 among sites increased significantly with plant age/time since transplant, consistent with the succession hypothesis; however, PCo2 showed the opposite pattern, indicating that root communities converged in this dimension as plants aged. **(b)** Along PCo1 and PCo2, root communities of experimental plants became more similar to those of endogenous plants as they aged, consistent with the succession hypothesis. **(c)** As field plants aged, their root communities diverged from those of greenhouse plants along PCo1 and PCo3 (consistent with the succession hypothesis) but showed the opposite pattern on PCo2.

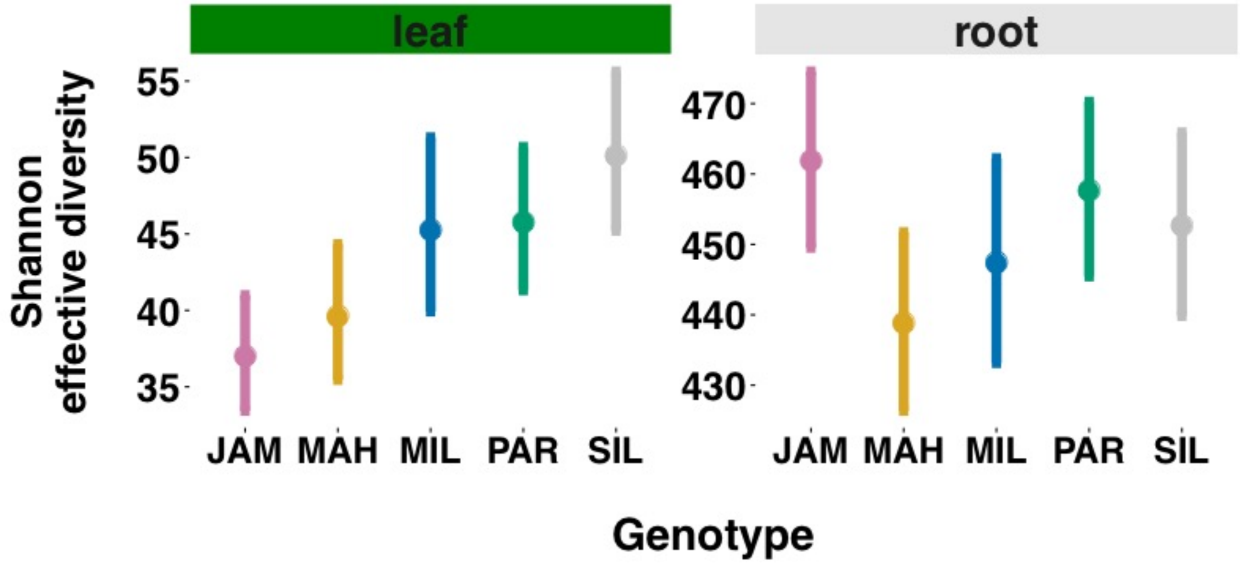

Supplementary Figure 12 | Microbiome evenness is robust to host genotype. Least-squares mean Shannon effective diversity ($e^{\text{Shannon entropy}}$) is plotted for each plant genotype in leaves and roots. ANOVA, P>0.05; detailed statistics are found in Table 1. Error bars show one standard error of the mean.

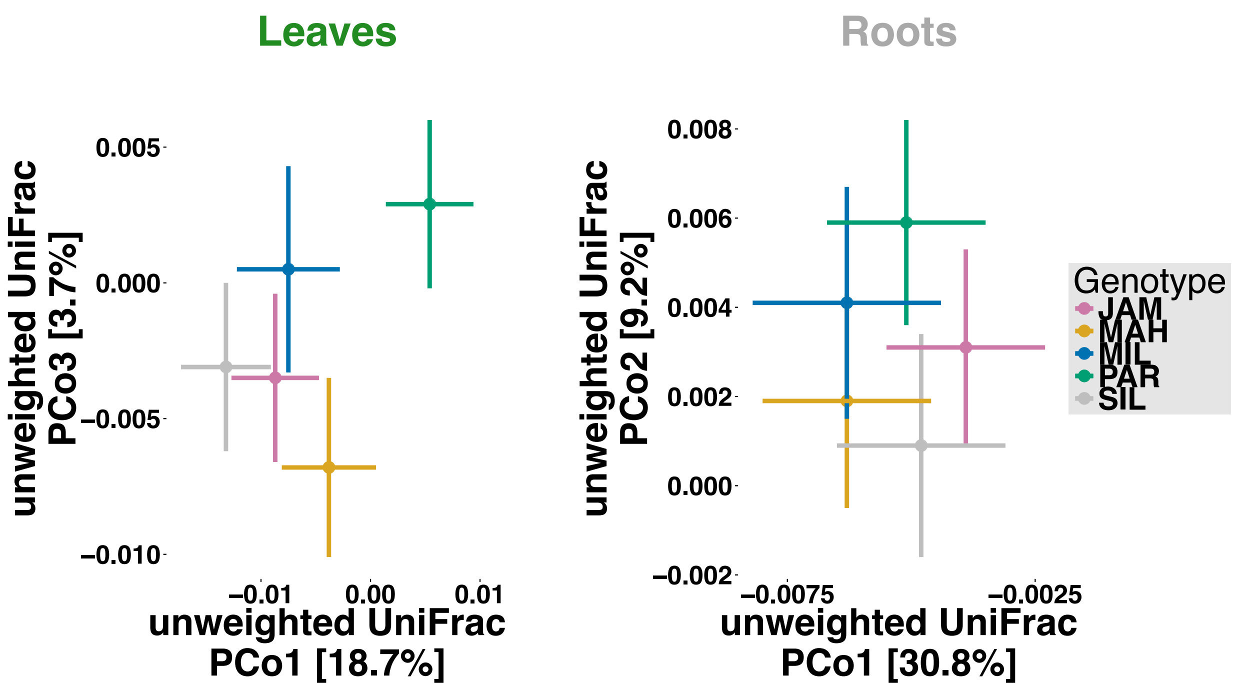

Supplementary Figure 13 I Bacterial community composition separates by host plant genotype in unweighted UniFrac ordination for **(a)** leaves (ANOVA, P<0.01), but not **(b)** roots (ANOVA, P>0.05). Least squares mean PCoA coordinates are plotted to highlight the influence of host genotype after controlling for other sources of variation using linear mixed effects models. Detailed statistics are found in Supplementary Table 5. Bars depict one standard error of the mean.

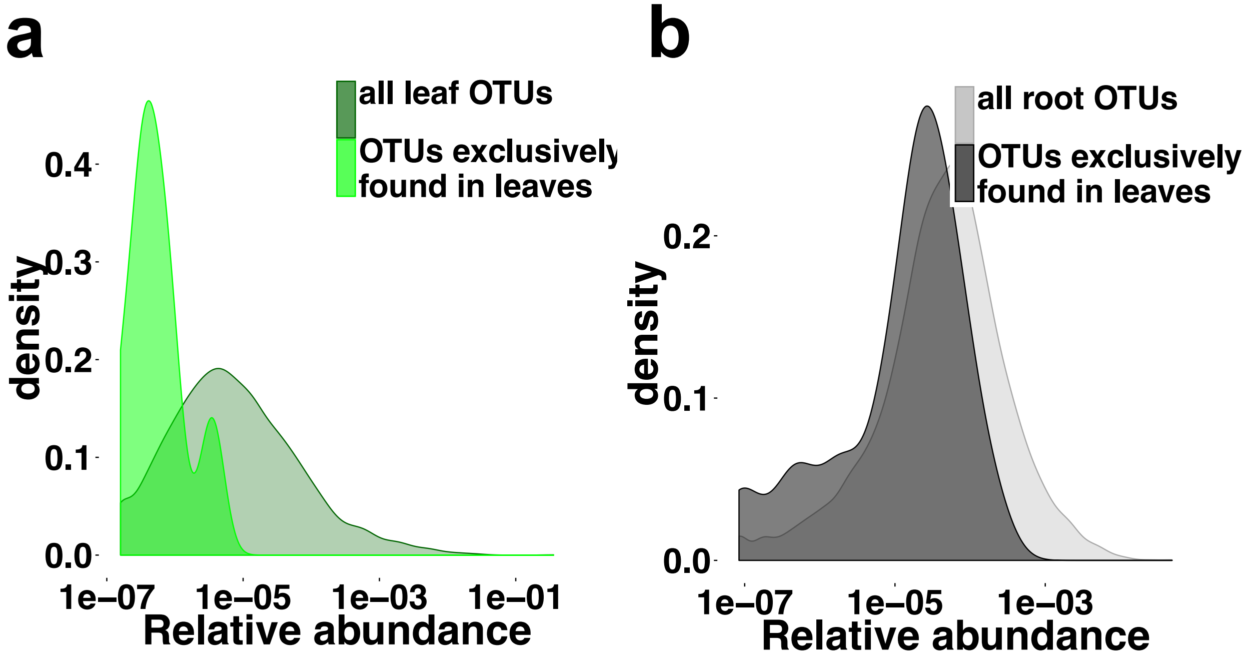

Supplementary Figure 14 | Organ-specific OTUs tended to be rare. (a) The 7 OTUs exclusively observed in leaves were rarer than average within leaf communities. **(b)** The 73 OTUs exclusively observed in roots were only slightly rarer than average within root communities.

Supplementary Table 1 l Sample sizes for each age group in each field site.

Site	Leaf Age=2	Leaf Age=3	Leaf Age=4	Root Age=2	Root Age=3	Root Age=4
Jam	27	42	15	29	45	15
Mah	31	56	29	30	58	29
Mil	19	24	**0**	21	27	**2**
Par	**0**	23	**0**	**0**	23	**1**
Sil	28	58	20	29	56	19

Sample sizes above include all surviving plants at Mil and Par.

Supplementary Table 2 | The 30 most abundant bacterial families in *Boechera stricta* leaves in three natural habitats with their relative abundances

Site: Jam	%	Site: Mah	%	Site: Sil	%
Sphingomonadaceae	24	Sphingomonadaceae	42.8	Sphingomonadaceae	48
Cytophagaceae	6.4	Cytophagaceae	12	Cytophagaceae	9.1
Microbacteriaceae	6	Sphingobacteriaceae	11.2	Microbacteriaceae	8.3
Sphingobacteriaceae	5.1	Microbacteriaceae	8.7	Sphingobacteriaceae	6.5
Enterobacteriaceae	4.8	Methylobacteriaceae	3.1	Methylobacteriaceae	5.1
Oxalobacteraceae	4.2	Nocardioidaceae	2.8	Nocardioidaceae	2.6
Pseudomonadaceae	2.9	Oxalobacteraceae	2.2	Pseudomonadaceae	2.3
Chitinophagaceae	2.9	Pseudomonadaceae	1.5	Acidobacteriaceae	2.2
Nocardioidaceae	2.7	Comamonadaceae	1.5	Chitinophagaceae	1.5
Chthoniobacteraceae	2.5	Chitinophagaceae	1.3	Comamonadaceae	1.5
Acidobacteriaceae	2.4	Patulibacteraceae	1	Oxalobacteraceae	1.4
Acetobacteraceae	2.1	Caulobacteraceae	0.9	Rhizobiaceae	1.1
Nocardiaceae	1.8	Acetobacteraceae	0.8	Patulibacteraceae	1
Gaiellaceae	1.8	Hyphomicrobiaceae	0.8	Acetobacteraceae	0.9
Geodermatophilaceae	1.8	Mycobacteriaceae	0.7	Aurantimonadaceae	0.7
Methylobacteriaceae	1.3	Aurantimonadaceae	0.7	Mycobacteriaceae	0.7
Comamonadaceae	1.3	Pseudonocardiaceae	0.6	Caulobacteraceae	0.6
Blattabacteriaceae	1.3	Rhizobiaceae	0.6	Kineosporiaceae	0.4
Micrococcaceae	1.3	Kineosporiaceae	0.4	Beijerinckiaceae	0.4
Bradyrhizobiaceae	1.3	Nocardiaceae	0.4	Bradyrhizobiaceae	0.3
Ellin5301	1	Solirubrobacteraceae	0.4	Bdellovibrionaceae	0.3
Solibacteraceae	1	Acidobacteriaceae	0.4	Cystobacterineae	0.3
Sporichthyaceae	1	Bradyrhizobiaceae	0.4	Geodermatophilaceae	0.3
Solirubrobacteraceae	0.9	Frankiaceae	0.3	Pseudonocardiaceae	0.3
Phormidiaceae	0.8	Nakamurellaceae	0.3	Chthoniobacteraceae	0.3
Caulobacteraceae	0.8	Xanthomonadaceae	0.2	Hyphomicrobiaceae	0.3
Hyphomicrobiaceae	0.8	Micromonosporaceae	0.2	Frankiaceae	0.2
Koribacteraceae	0.7	Chthoniobacteraceae	0.2	Xanthomonadaceae	0.2
Rhizobiaceae	0.7	Ellin6075	0.2	Williamsiaceae	0.2
Patulibacteraceae	0.7	Geodermatophilaceae	0.2	Methylocystaceae	0.2

Supplementary Table 3 | The 30 most abundant bacterial families in *Boechera stricta* roots in three natural habitats with their relative abundances

Site: Jam	%	Site: Mah	%	Site: Sil	%
Bradyrhizobiaceae	8.8	Comamonadaceae	9	Bradyrhizobiaceae	8.5
Nocardioidaceae	5.3	Bradyrhizobiaceae	7.7	Comamonadaceae	7.5
Comamonadaceae	5.1	Microbacteriaceae	6.6	Microbacteriaceae	4.9
Microbacteriaceae	4.9	Sinobacteraceae	5	Chitinophagaceae	4.8
Sphingomonadaceae	4.4	Hyphomicrobiaceae	4.6	Hyphomicrobiaceae	4.8
Opitutaceae	4.2	Chitinophagaceae	4	Sphingomonadaceae	4.5
Hyphomicrobiaceae	3.9	Sphingomonadaceae	4	Nocardioidaceae	4.3
Streptomycetaceae	3.9	Cytophagaceae	3.6	Sinobacteraceae	4
Chitinophagaceae	3.5	Xanthomonadaceae	3.4	Chthoniobacteraceae	2.9
uncl. Actinomycetales	3.4	Nocardioidaceae	3.2	Cytophagaceae	2.8
Sphingobacteriaceae	3.2	Micromonosporaceae	2.8	Ellin5301	2.7
Ellin5301	2.8	Kineosporiaceae	2.5	Ellin6075	2.3
Pseudonocardiaceae	2.7	Caulobacteraceae	2.4	Caulobacteraceae	2.3
Xanthomonadaceae	2.7	Chthoniobacteraceae	2.2	Opitutaceae	2.2
Cytophagaceae	2.4	Ellin5301	2	Gemmataceae	2
Caulobacteraceae	2.4	Streptomycetaceae	2	Xanthomonadaceae	1.9
Actinosynnemataceae	2.3	Rhizobiaceae	1.8	A4b	1.8
Micromonosporaceae	2	A4b	1.7	Streptomycetaceae	1.8
Rhizobiaceae	1.8	Ellin6075	1.6	Micromonosporaceae	1.6
Oxalobacteraceae	1.7	Opitutaceae	1.6	Pirellulaceae	1.5
Sinobacteraceae	1.6	Oxalobacteraceae	1.6	Sphingobacteriaceae	1.5
Mycobacteriaceae	1.6	Gemmataceae	1.5	Haliangiaceae	1.3
Rhodospirillaceae	1.5	Sphingobacteriaceae	1.4	Rhizobiaceae	1.2
Pirellulaceae	1	Pirellulaceae	1.3	Rhodospirillaceae	1.2
Pseudomonadaceae	1	Rhodospirillaceae	1.2	Solirubrobacteraceae	1.1
Kineosporiaceae	1	Haliangiaceae	1	Kineosporiaceae	1.1
Phyllobacteriaceae	1	Solirubrobacteraceae	1	Pseudomonadaceae	1.1
Gemmataceae	1	Rhodocyclaceae	1	Acidobacteriaceae	1
Chthoniobacteraceae	0.9	Pseudomonadaceae	0.9	EB1017	0.9
Ellin6075	0.8	Pseudonocardiaceae	0.7	Solibacteraceae	0.9

Uncl. = unclassified at the family level

Supplementary Table 4 | The 30 most abundant bacterial families in bulk soils from three natural *Boechera stricta* habitats with their relative abundances

Site: Jam	%	Site: Mah	%	Site: Sil	%
Chthoniobacteraceae	22.2	Chthoniobacteraceae	19.3	Chthoniobacteraceae	10.7
Koribacteraceae	11.1	Bradyrhizobiaceae	11.1	Hyphomicrobiaceae	8.9
Gaiellaceae	7.9	Gaiellaceae	8.8	Bradyrhizobiaceae	8.5
Gemmataceae	5.5	Hyphomicrobiaceae	6.8	Gaiellaceae	6.4
Bradyrhizobiaceae	5.1	Gemmataceae	4.3	Gemmataceae	6
Hyphomicrobiaceae	3.7	Koribacteraceae	3.1	Chitinophagaceae	3.7
Chitinophagaceae	2.6	Chitinophagaceae	3	Pirellulaceae	3.1
Solibacteraceae	2.5	Sphingomonadaceae	2.7	Solibacteraceae	3.1
Sphingomonadaceae	2.5	EB1003	2	Ellin515	2.9
Nitrososphaeraceae	1.9	Ellin5301	1.5	Ellin5301	2.3
EB1003	1.8	Solirubrobacteraceae	1.5	Cytophagaceae	2.2
Ellin5301	1.8	Solibacteraceae	1.5	Opitutaceae	2.1
Pirellulaceae	1.3	Pirellulaceae	1.4	Acidobacteriaceae	2.1
Ellin515	1.3	Sinobacteraceae	1.4	EB1017	2
Sphingobacteriaceae	1	EB1017	1.3	Sinobacteraceae	2
Ellin6075	1	Micromonosporaceae	1.2	Sphingomonadaceae	1.7
Pseudonocardiaceae	1	Mycobacteriaceae	1.2	Comamonadaceae	1.6
Nitrospiraceae	0.9	Opitutaceae	1.1	Koribacteraceae	1.6
Isosphaeraceae	0.9	Nocardioidaceae	1.1	Isosphaeraceae	1.6
Nocardioidaceae	0.9	Comamonadaceae	1	Nocardioidaceae	1.5
Sinobacteraceae	0.8	Isosphaeraceae	1	Rhodospirillaceae	1.5
Solirubrobacteraceae	0.8	Ellin6075	0.9	EB1003	1.1
Rhodospirillaceae	0.8	Rhodospirillaceae	0.9	Ellin6075	1.1
Opitutaceae	0.7	Microbacteriaceae	0.8	Mycobacteriaceae	1
Uncl. Pedosphaerales	0.7	Syntrophobacteraceae	0.8	auto67_4W	0.9
Oxalobacteraceae	0.7	Uncl. Gemmatimonadales	0.7	Solirubrobacteraceae	0.9
Comamonadaceae	0.7	Cytophagaceae	0.7	Xanthomonadaceae	0.8
Microbacteriaceae	0.6	C111	0.7	Planctomycetaceae	0.8
Acidobacteriaceae	0.6	Uncl. Bacillales	0.7	Microbacteriaceae	0.8
Myxococcaceae	0.6	Acidobacteriaceae	0.7	Coxiellaceae	0.8

Uncl. = unclassified at the family level

Supplementary Table 5 I Experimental factors predicting bacterial presence-absence in the leaves and roots of *Boechera stricta*

	Leaf			Root		
	**PCo1**	**PCo2**	**PCo3**	**PCo1**	**PCo2**	**PCo3**
R^2	0.85	0.80	0.87	0.98	0.96	0.91
Site	$F_{2,62}$=262 **P<3e^{-16}**	$F_{2,50}$=1.8 P=0.18	$F_{2,208}$=573 **P<3e^{-16}**	$F_{2,31}$=1544 **P<3e^{-16}**	$F_{2,35}$=993 **P<3e^{-16}**	$F_{2,33}$=11.2 **P=0.00019**
Geno.	$F_{4,258}$=5.5 **P=0.00082**	$F_{4,264}$=1.7 P=0.29	$F_{4,13}$=1.6 P=0.29	$F_{4,32}$=0.5 P=1	$F_{4,36}$=0.8 P=1	$F_{4,259}$=0.6 P=1
Geno. x Site	$F_{8,257}$=2.8 **P=0.016**	$F_{8,263}$=0.9 P=1	$F_{8,274}$=0.6 P=1	$F_{8,253}$=1.5 P=0.43	$F_{8,253}$=1.4 P=0.43	$F_{8,257}$=0.4 P=0.92
Age	$F_{2,69}$=2.8 P=0.14	$F_{2,68}$=4.4 **P=0.047**	$F_{2,272}$=1.5 P=0.23	$F_{2,53}$=1.4 P=0.27	$F_{2,58}$=36.6 **P=1.1e^{-10}**	$F_{2,57}$=449 **P<3e^{-16}**
Age x Site	$F_{4,67}$=1.4 P=0.49	$F_{4,67}$=1.8 P=0.42	$F_{4,271}$=0.4 P=0.8	$F_{4,53}$=2.2 P=0.076	$F_{4,57}$=6.0 **P=0.00084**	$F_{4,57}$=16.2 **P=2.1e^{-08}**
Year	$F_{1,114}$=4.6 P=0.066	$F_{1,61}$=10.3 **P=0.0064**	$F_{1,57}$=1.3 P=0.25	$F_{1,52}$=0.5 P=0.5	$F_{1,67}$=22.4 **P=2.4e^{-05}**	$F_{1,57}$=345 **P<3e^{-16}**
Year x Site	$F_{2,95}$=2.0 P=0.27	$F_{2,76}$=0.3 P=0.75	$F_{2,188}$=2.9 P=0.18	$F_{2,48}$=1.4 P=0.25	$F_{2,61}$=9.8 P=**6e^{-04}**	$F_{2,55}$=7.2 **P=0.0033**
Block	χ^2_1=3.3 P=0.21	χ^2_1=3.0 P=0.21	χ^2_1=2.3e^{-13} P=1	χ^2_1=20.7 **P=1.6e^{-05}**	χ^2_1=10.6 **P=0.0012**	χ^2_1=13.4 **P=5e^{-04}**
Line	χ^2_1=2.3e^{-13} P=1	χ^2_1=2.3e^{-13} P=1	χ^2_1=0.0 P=1	χ^2_1=1.9 P=0.34	χ^2_1=4.6 P=0.094	χ^2_1=4.5e-13 P=1
logObs	$F_{1,273}$=14.1 **P=0.0004**	$F_{1,256}$=360 **P<3e^{-16}**	$F_{1,275}$=2.7 P=0.1	$F_{1,148}$=12.6 **P=0.002**	$F_{1,103}$=0.1 P=1	$F_{1,147}$=0.0 P=1
MiSeq run	χ^2_1=14.2 **P=0.0005**	χ^2_1=0.0 P=0.95	χ^2_1=0.9 P=0.68	χ^2_1=0.2 P=1	χ^2_1=0.0 P=1	χ^2_1=0.0 P=1

Statistics describe linear random-intercept models of unweighted UniFrac principal coordinates in leaves and roots. All p-values were adjusted for multiple comparisons using the sequential Bonferroni correction. Significance was assessed using Type III ANOVA with F-tests for fixed effects and likelihood ratio tests for random effects.

Supplementary Table 6 I Experimental factors predicting glucosinolate content of leaves and roots

	Leaf	Root	
	**BC-ratio** R^2=0.95	**BC-ratio** R^2=0.44	**Total [GLS]** R^2=0.39
**Site**	$F_{2,7}$=0.78 P=0.49	$F_{4,77}$=2.88 **P=0.028**	$F_{4,50}$=2.79 **P=0.036**
**Genotype**	$F_{4,32}$=8.30 **P=0.0001**	$F_{4,74}$=1.13 P=0.35	$F_{4,79}$=0.39 P=0.81
**Genotype x Site**	$F_{8,54}$=4.39 **P=0.0004**	$F_{16,262}$=0.47 P=0.96	$F_{16,263}$=0.69 P=0.80
**Age**	$F_{1,52}$=2.77 P=0.10	$F_{1,259}$=0.64 P=0.42	$F_{1,40}$=0.11 P=0.74
**Age x Site**	$F_{1,52}$=0.14 P=0.71	$F_{2,256}$=0.37 P=0.69	$F_{2,40}$=0.87 P=0.43
**Block**	X^2_1=0 P=1	X^2_1=0 P=1	X^2_1=6.01 **P=0.01**
**Line**	X^2_1=43.02 **P=5e^{-11}**	X^2_1=17.8 **P=3e^{-5}**	X^2_1=5.07 **P=0.02**
**HPLC batch**	X^2_1=0.345 P=0.6	X^2_1=44.8 **P=2e^{-11}**	X^2_1=5.99 **P=0.01**

Linear random-intercept models of glucosinolate profiles in leaves and roots. “BC-ratio,” a metric of glucosinolate quality, was arcsine-square root transformed before analysis. Total [GLS] = total concentration of glucosinolates (µmol per mg dry tissue). Total [GLS] was square-root transformed before analysis. “HPLC batch” is a nuisance variable to control for noise attributed to differences among HPLC runs. Significance was assessed using Type III ANOVA with F-tests for fixed effects and likelihood ratio tests for random effects.

Supplementary Table 7 l The 30 most abundant bacterial families in *Boechera stricta* roots grown in field environments and in potting soil in the greenhouse, as well as the most abundant families in bulk potting soil, are provided with their relative abundances.

Roots: 5 field sites	%	Roots: Greenhouse	%	Bulk potting soil	%
Comamonadaceae	10.8	Comamonadaceae	22.8	Ktedonobacteraceae	9.7
Bradyrhizobiaceae	7.2	Cytophagaceae	6.2	Hyphomicrobiaceae	6.6
Chitinophagaceae	4.2	Rhizobiaceae	5.5	Xanthomonadaceae	5.5
Sphingomonadaceae	4.1	Xanthomonadaceae	5.5	Opitutaceae	5.5
Microbacteriaceae	3.9	Caulobacteraceae	4.7	Streptomycetaceae	5.1
Hyphomicrobiaceae	3.9	Chitinophagaceae	4.5	Chitinophagaceae	3.6
Nocardioidaceae	3.7	Oxalobacteraceae	4.1	Comamonadaceae	3.4
Cytophagaceae	3.4	Rhodospirillaceae	3.7	Rhodospirillaceae	3.4
Sinobacteraceae	3.1	Bradyrhizobiaceae	3.6	Thermomonosporaceae	3.4
Xanthomonadaceae	2.7	Hyphomicrobiaceae	2.8	Sphingomonadaceae	3.2
Caulobacteraceae	2.5	Sphingomonadaceae	2.3	Caulobacteraceae	3.1
Opitutaceae	2.5	Opitutaceae	1.8	Bradyrhizobiaceae	2.7
Streptomycetaceae	2.4	Fimbriimonadaceae	1.6	Ellin517	2.7
Oxalobacteraceae	2.2	Rhodocyclaceae	1.6	Microbacteriaceae	2.3
Micromonosporaceae	2.2	Erythrobacteraceae	1.5	Pirellulaceae	2.3
Ellin5301	2.1	Streptomycetaceae	1.5	Phormidiaceae	2
Rhizobiaceae	1.9	Sinobacteraceae	1.4	Sinobacteraceae	1.8
Chthoniobacteraceae	1.9	Chthoniobacteraceae	1.2	Nocardiaceae	1.7
Rhodocyclaceae	1.9	Microbacteriaceae	1.2	Gemmataceae	1.7
Sphingobacteriaceae	1.8	Verrucomicrobiaceae	1.1	Pseudanabaenaceae	1.7
Rhodospirillaceae	1.4	Nocardioidaceae	1.1	Nocardioidaceae	1.6
A4b	1.4	Micromonosporaceae	1	Rhizobiaceae	1.5
Ellin6075	1.4	Pirellulaceae	1	Fimbriimonadaceae	1.3
uncl. Actinomycetales	1.3	Pseudomonadaceae	0.9	Chthoniobacteraceae	1.3
Kineosporiaceae	1.3	Alteromonadaceae	0.9	Mycobacteriaceae	1.2
Gemmataceae	1.3	Kouleothrixaceae	0.9	Nostocaceae	1
Pirellulaceae	1.2	Gemmataceae	0.8	Isosphaeraceae	0.9
Pseudonocardiaceae	1	Phyllobacteriaceae	0.8	Gomphosphaeriaceae	0.8
Haliangiaceae	1	Sphingobacteriaceae	0.8	Solibacteraceae	0.8
Pseudomonadaceae	0.9	Methylophilaceae	0.7	Verrucomicrobiaceae	0.7

Uncl. = unclassified at the family level

Supplementary Note 1 l Genetic variation and plasticity of glucosinolate content could partially underlie patterns of microbiome variation.

For a subset of plants harvested in 2011, we also quantified glucosinolates (GLS), secondary chemicals produced by *B. stricta* to protect against predators[1]. Because GLS are known to influence plant-associated bacterial communities and microbial pathogens[2-3], variation in GLS caused by genetic effects or phenotypic plasticity could partially underlie patterns of microbiome variation. To test this hypothesis we measured "BC-ratio," (the proportion of aliphatic GLS derived from branched-chain amino acids), which is a measure of GLS quality that affects biological activity[1,4]; and in roots we also measured absolute concentration of GLS. We modeled GLS using the same predictors that we used to model microbiome features except for Year Harvested, because all GLS data was collected in a single year (2011).

Leaf BC-ratio varied strongly among genotypes and also showed a genotype-by-site interaction (Supplementary Fig. 8, Supplementary Table 6), consistent with the hypothesis that GLS quality partially underlies differential abundance of leaf-associated bacteria between genotypes depending on site (Fig. 6; Fig. 7). Leaf BC-ratio did not differ by site when averaged across all genotypes (Supplementary Table 6).

In the current study both absolute GLS concentration and BC-ratio in roots varied among gardens, suggesting that if root-associated microbes are sensitive to GLS (as reported in other systems[3]), the strong differences in bacterial communities among sites (Fig. 2) may have been caused not only by biogeographic patterns, but also by plasticity of GLS production by the plant (Supplementary Table 6, Supplementary Fig. 8).

Furthermore, both root BC-ratio and total glucosinolate concentration varied among lines within genotypes, offering one plausible mechanism for the modest heritability of root bacteria measured in this study (Supplementary Table 6, Supplementary Fig. 8).

Methods for glucosinolate analysis. During the 2011 sample collection, we simultaneously subsampled leaves and roots for analysis of glucosinolate profiles. A small amount (roughly ~30 mg fresh weight) of tissue was submerged immediately in 70% methanol. Because of resource limitations we only subsampled 98 rosettes (from Jackass Meadow, Parker Meadow, and Mahogany Valley only) and 306 roots (from all five gardens). Tissue samples were submerged in 70% methanol for a minimum of one month and randomized into 96-well plates. To extract glucosinolates, the resulting leachate was passed through a column of 35mg DEAE-Sephadex A-25 chloride (Sigma Aldrich, St. Louis, USA) that had been equilibrated with 20mM sodium acetate for one hour. To each column we added 50µL 1mM sinigrin (Sigma Aldrich, St. Louis, USA) as an internal standard. For leaf samples we washed columns with 70% methanol (750µL, twice), diH_2O (750µL, twice), 20mM NaOAc (750µL, once), and diH_2O (750µL, twice); for root samples we washed columns with 70% methanol (450µL, once), diH_2O (450µL, once), 1M NaOAc (300µL, once), and 20mM NaOAc (150µL, once). We then added 30µL of aqueous sulfatase and incubated the columns overnight before eluting the desulfinated glucosinolates in 70% HPLC-grade methanol (75µL, twice) and HPLC-grade water (75µL, twice).

Fifty µL of each extract was analyzed on an Agilent 1100-series high-performance liquid chromatography machine with a diode array detector and a Zorbax Eclipse XDB-

C18 column (4.6 x 150 mm, 5-micron pore size; Agilent Technologies, Santa Clara, USA). We separated glucosinolates using a water-acetonitrile (ACN) gradient at 40°C and a flow rate of 1mL/minute: [ACN] was held at 1.5% for 6 minutes then increased to 2.5% by minute 8, to 5% by minute 15, to 18% by minute 17, to 46% by minute 23, to 92% by minute 24, and then decreased to 1.5% by minute 29. We identified compounds based on UV absorption spectrum at 229 nm and retention time[5]. We calculated the absolute amount of each compound by multiplying the area under its HPLC peak by the known amount of sinigrin (0.05 µmol), and then dividing by the area under the sinigrin peak and by its relative response factor[6]. We calculated "BC-ratio" as the proportion of aliphatic glucosinolates derived from branched-chain amino acids[1,4]. For roots only we also calculated total glucosinolate concentration by summing absolute amounts of all compounds and dividing by the dry mass of the tissue sample. Because we did not weigh leaf samples, we could not standardize by tissue mass, and therefore we report only BC-ratio for rosettes. BC-ratio was arcsine-square root transformed and Total [GLS] was square-root transformed to improve homoscedasticity. We fit linear mixed models with fixed effects Site + Genotype + Site*Genotype + Age + Site*Age and random intercepts Block + Line + HPLC batch. We then assessed statistical significance of fixed predictors using Type III ANOVA with Satterthwaite's approximation of denominator degrees of freedom in the package lmerTest[7], and of random effects using likelihood ratio tests.

Supplementary Note 2 | Age-related changes in root bacterial communities are partially, but not completely, consistent with a hypothesis of ongoing succession after transplant from greenhouse.

In fall 2011, we planted a small greenhouse replicate experiment with a subset of the genetic lines (4 lines per genotype) used in the field experiment. Seedlings were planted into the same brands of standard greenhouse potting soil that were used to grow rosettes for the field experiment, and watered with tap water (see main Methods). Although there is no guarantee that the resident potting-soil bacterial community (or the tap water community) was exactly the same in 2011 as in 2008 or 2009, we assume here that it is an approximate replication of the initial inoculum for roots of our experimental plants. Roots were harvested from 6-week-old rosettes—the same developmental stage as the field experimental plants at the time of transplant—and processed along with the samples from the field. We summarized root community composition using unconstrained principal coordinates analysis of weighted UniFrac distances between OTU tables (after applying the variance-stabilizing transformation) for all root samples for which data were available, as described in the main Methods. The dissimilarity between roots growing in potting soil and roots transplanted into the field dwarfed the dissimilarity between the three field sites (Supplementary Figure 10; Supplementary Table 7), which was by far the strongest source of variation in our field experiment (see main manuscript, Tables 1-2, Figs. 2-3).

If community turnover after transplant from greenhouse to field is the only mechanism of the observed effects of host age on root microbiomes, then we would

expect to observe the following patterns: (i) As plant age increases, the average root communities at the three field sites should become more different from each other, because they have had more time to diverge from the common potting-soil inoculum; (ii) As plant age increases, root communities of experimental plants should become more similar to those of local endogenous plants; and (iii) As plant age increases, root communities of plants in the field should become less similar to those of plants grown in potting soil in the greenhouse, which represent the original common inoculum of the field plants.

To test these predictions, we compared the means of the three major PCoA axes for each age group in each site. To control for other known sources of variation (including host genotype, year of observation, block within field site, and technical nuisance variables), we used least-squares means from the Age x Site interaction term in the statistical models described in the main text. Considering the effect of age independently in each site allowed for communities to change in different directions in PCoA space without obscuring results; for instance, root communities might diverge from the potting-soil inoculum in the direction of positive PCo1 at one site, but in the direction of negative PCo1 in another; yet both are becoming more distinct from the potting-soil control. From the least-squares means of each age group at each site, for each of PCo1, PCo2, and PCo3 we calculated (i) the variance among sites; (ii) the absolute value of the distance from the mean endogenous root community at each site; and (iii) the absolute value of the distance from the mean potting-soil root community.

Each of these values was regressed onto plant age; the slopes of these regressions describe the movement of root communities in PCoA space as host plants age.

We tested the significance of each of these slopes using permutation tests. Age groups were permuted among all experimental plants; other metadata (genotype, year, block, etc.) and PCoA position were held constant. For each of 999 permuted datasets, the linear mixed model was re-fitted and the parameters (i), (ii), and (iii) described above were re-calculated. The resulting distributions describe the expected parameter values under the null hypotheses that (i) variance among sites is not related to host age/time since transplant; (ii) similarity to endogenous plants is not related to host age/time since transplant; and (iii) similarity to greenhouse plants is not related to host age/time since transplant.

The effect of plant age on root PCo1 rejects the null hypothesis for all three predictions, indicating that this axis of variation likely describes a component of the root microbiome that is still gradually changing simply due to dilution of the initial potting-soil inoculum (Supplementary Figure 11). In contrast, both PCo2 and PCo3 only reject the null hypothesis for one of the three predictions. In fact, PCo2 shows two significant trends in the *opposite* direction: along this axis of variation, the mean root communities at the three sites became *more* similar with time since transplant (Supplementary Figure 11a), and *more* similar to the root communities of greenhouse-grown plants (Supplementary Figure 11b). These results suggest that the ongoing replacement of potting-soil inoculum by wild bacteria after transplant is not the sole cause of the observed changes in root community composition attributed to plant age.

Detailed methods for greenhouse sub-experiment. Surface-sterilized seeds (1-minute wash and vortex in 70% ethanol + 0.1% Triton X-100; vortex and 15-minute incubation in 10% bleach + 0.1% Triton X-100; three rinses with sterile diH_2O) were plated on autoclaved filter paper in petri dishes moistened with sterile diH_2O, stratified in the dark at 4°C for one week, and then allowed to germinate in a growth chamber (22°C, ambient humidity, 11-hour day length). One week after germination, seedlings were transplanted into one of five soils: standard greenhouse potting soil (as described in main Methods) or wild soil collected from the field site Mah, Mil, Par, or Sil. In this analysis we focus only on the plants grown in potting soil, because we are specifically interested in replicating the starting conditions for the plants that were later moved into the field. All seedlings grew for 6 weeks in a greenhouse with conditions as described in the main Methods. Plants were watered with tap water as needed. We harvested roots and removed soil particles using flame-sterilized utensils, and rinsed them with sterilized diH_2O. These greenhouse-grown roots (total N=162) as well as samples of unplanted bulk soils (N=23) were then processed along with the samples from the field (see main Methods).

Supplementary References

1. Prasad, K. V. S. K. *et al.* A gain-of-function polymorphism controlling complex traits and fitness in nature. *Science* **337,** 1081–1084 (2012).

2. Witzel, K. *et al.* Verticillium suppression is associated with the glucosinolate composition of Arabidopsis thaliana leaves. *PLoS ONE* **8,** e71877 (2013).

3. Bressan, M. *et al.* Exogenous glucosinolate produced by Arabidopsis thaliana has an impact on microbes in the rhizosphere and plant roots. *ISME J* **3,** 1243–1257 (2009).

4. Schranz, M. E., Manzaneda, A. J., Windsor, A. J., Clauss, M. J. & Mitchell-Olds, T. Ecological genomics of Boechera stricta: identification of a QTL controlling the allocation of methionine-vs branched-chain amino acid-derived glucosinolates and levels of insect herbivory. *Heredity* **102,** 465–474 (2009).

5. Olson-Manning, C. F., Lee, C.-R., Rausher, M. D. & Mitchell-Olds, T. Evolution of flux control in the glucosinolate pathway in Arabidopsis thaliana. *Molecular Biology and Evolution* **30,** 14–23 (2012).

6. Clarke, D. B. Glucosinolates, structures and analysis in food. *Analytical Methods* **2,** 310 (2010).

7. Kuznetsova, A., Brockhoff, P. B. & Christensen, R. H. B. *lmerTest: Tests in Linear Mixed Effects Models.* (2015).
